# Electronic Delocalization of Fe Atom–Cluster for Long-Term Stable Electromagnetic Wave Absorption in Marine Environments

**DOI:** 10.1007/s40820-026-02210-y

**Published:** 2026-05-11

**Authors:** Shaocong Zhong, Xinyu Wang, Rurong Zou, Chang Long, Pianpian Zhang, Xueting Zhang, Zihao Zhao, Ying Liu, Can Cui, Yanan Yang, Long Xia

**Affiliations:** 1 College of Materials Science and Engineering, Harbin Institute of Technology (Weihai), Weihai, 264209 People’s Republic of China; 2 Aerospace Science and Industry Wuhan Magnetism-Electron Co., Ltd, Wuhan, 430074 People’s Republic of China

**Keywords:** Fe single atom, Fe cluster, Electromagnetic wave absorption, Electronic delocalization, Marine environment

## Abstract

High‐performance electromagnetic wave (EMW) absorbers with environmental adaptability are essential for advanced maritime stealth and electromagnetic protection. Herein, we design a synergistic absorber integrating Fe clusters (Fe_AC_) and single atoms (Fe_SA_) to tackle the issue of EMW attenuation under high-salinity and humidity marine conditions. First-principles calculations and experiments reveal that Fe_AC_ and Fe_SA_ anchored on a *π*‐conjugated carbon support form a delocalized electronic space that enables long-range electronic interactions and multicenter synergistic coupling. This electronic synergy markedly strengthens conduction loss and dipolar polarization, delivering a minimum reflection loss of − 68.78 dB and an effective absorption bandwidth of 6.00 GHz at a low loading of 6 wt%. Notably, Fe_AC_ exhibits a thermodynamically preferred adsorption toward Cl^−^, generating locally enriched negative‐charge regions that mitigate direct ionic attack on atomically dispersed Fe_SA_ sites and thereby suppress corrosion-induced performance degradation. In addition, the absorber film demonstrates mechanical flexibility and thermal insulation, highlighting its potential for durable and high‐efficiency maritime EMW protection applications.

**Supplementary Information:**

The online version contains supplementary material available at 10.1007/s40820-026-02210-y.

## Highlights

- Fe cluster–single-atom synergy induces electron delocalization and charge redistribution, enabling multicenter coupling for enhanced electromagnetic wave attenuation.
- The synergistic electronic regulation significantly strengthens dielectric loss and conduction loss, leading to high‐efficiency electromagnetic wave absorption at low filler loadings.
- Fe clusters preferentially adsorb Cl^−^, generating localized negatively charged regions that mitigate ionic attack on single-atom sites and improve durability in marine conditions.

**Supplementary Information:**

The online version contains supplementary material available at 10.1007/s40820-026-02210-y.

## Graphical abstract

**Figure:**
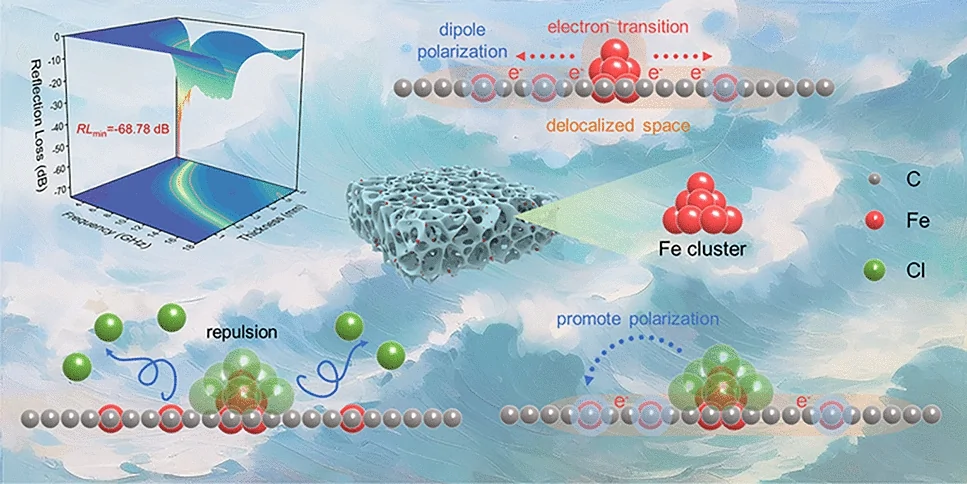

## Introduction

With the rapid development of wireless communication, marine exploration, and radar detection technologies, the escalating complexity of the marine electromagnetic environment has become a pivotal challenge restricting the performance of maritime systems [1, 2]. In particular, stealth technologies that evade radar detection are indispensable for augmenting the survivability and strategic deterrence of naval vessels and maritime aircraft, thereby necessitating the development of high‐performance electromagnetic wave (EMW) absorbing coatings [3]. Distinct from terrestrial scenarios, marine service environments are characterized by high humidity and salinity, imposing stringent requirements on the long-term functional stability of EMW absorbers and their ability to maintain effective radar cross-section (RCS) suppression under harsh conditions [4, 5].

In recent years, EMW absorbers have evolved from conventional magnetic-metal to carbon, polymers, 2D materials, and multiscale composites, enabling notable progress in impedance matching and multi-mechanism loss coupling. Nevertheless, simultaneously achieving “thin, lightweight, broadband, and strong” absorption remains difficult, mainly due to low active-site utilization and limited structural/interfacial tunability, which constrain both absorption efficiency and rational design. In this context, single-atom (SA) absorbers have attracted significant attention owing to their maximum atomic utilization, well-defined coordination structures, and tunable electronic properties [6–8]. Atomically dispersed active sites facilitate strong dipole polarization and efficient dielectric loss, offering a promising pathway toward lightweight and high-efficiency absorbers [9–12]. However, the highly localized electronic states associated with isolated atoms, while beneficial for polarization loss, inevitably limit long-range charge transport and electron hopping processes, thereby restricting the synergistic activation of multiple EMW loss mechanisms. Moreover, under marine environments, isolated atoms are vulnerable to aggregation and competitive coordination with aggressive Cl^−^, leading to severe poisoning of SA sites, substantially limiting SA absorber practical application in marine scenarios [13–16]. To enhance the stability of SA absorbers, multiple anti-corrosion strategies have been proposed, including protective encapsulation, defect engineering, and alloying. Despite the extensive efforts devoted to stabilizing single-atom absorbers, it should be noted that most existing strategies fundamentally rely on passive protection of isolated sites [17, 18]. Specifically, protective encapsulation suppresses moisture and ion attack but often compromises impedance matching and dielectric loss efficiency, while defect engineering mitigates corrosive ion adsorption at the expense of disrupting conductive networks [19–25]. Alloying can improve chemical stability, yet sacrifice atomic utilization and undermine the intrinsic advantage of atomic dispersion [26, 27]. As a result, such passive stabilization strategies inherently constrain charge transport and polarization dynamics, making it increasingly difficult to simultaneously realize environmental robustness and high electromagnetic energy dissipation. Under marine conditions dominated by aggressive Cl^−^ species, the degradation of SA absorbers is not governed solely by local coordination instability, but also by the absence of effective charge redistribution pathways capable of diverting corrosive ions away from atomically dispersed active sites. This intrinsic limitation suggests that stabilization of single atoms alone may be insufficient to overcome the long-term corrosion performance trade-off. Motivated by these considerations, introducing auxiliary functional units that can actively regulate local electronic environments and ion adsorption behavior emerges as a rational strategy to reconcile efficient EMW attenuation with environmental durability.

Herein, we report a Fe atomic cluster (Fe_AC_)–Fe single-atom (Fe_SA_) synergistic absorber (NC-Fe_AC2_) designed to enhance EMW dissipation through multiscale electronic cooperation, while simultaneously exhibiting improved tolerance toward moisture and marine corrosion [28, 29]. Anchoring Fe_SA_ in proximity to preferentially active Fe_AC_ enables electronic modulation via charge delocalization, thereby reinforcing local structural stability and facilitating electron hopping within the conductive network by lowering interfacial charge transfer barriers. Notably, Fe_AC_ exhibits a thermodynamic preference for Cl^−^ adsorption, enabling preferential capture of Cl^−^ and generating locally enriched negatively charged regions, and this repulsion effect effectively shields single-atom sites from direct Cl^−^ attack [30]. Unlike conventional passive protection strategies, the AC-SA synergistic strategy provides active protection by redistributing charges and selectively binding corrosive ions. Consequently, it preserves atomic dispersion and activity while harmonizing polarization and conductive loss mechanisms to enhance EM energy dissipation. This work deepens the understanding of the interplay between electronic synergy, EMW absorption performance, and environmental stability in SA-based absorbers and offers a feasible design paradigm for next-generation EMW absorbing materials.

## Experimental Section

### Materials

Dopamine hydrochloride (C_8_H_11_NO_2_‧HCl, 98%), sodium chloride (NaCl, 99.5%), iron(II) chloride tetrahydrate (FeCl_2_‧4H_2_O, 98%), hydrochloric acid (HCl, 36–38%), and ethyl alcohol (CH_3_CH_2_OH, 99.8%) were both purchased from Shanghai Maclin Biochemical Technology Co., Ltd (Shanghai, China).

### Preparation of NC, NC-Fe_ACX_ and NC-Fe_NP_

#### Preparations of the NC-Fe_ACX_

A mixture of 5.0 g of NaCl, 0.5 g of dopamine hydrochloride, and 0.2 g of FeCl_2_‧4H_2_O was dissolved in 100 mL of deionized water and stirred for 24 h to obtain a homogeneous solution. The above solution was then frozen with liquid nitrogen and freeze-dried at − 60 °C and 0.1 Pa for 3 days. The as-prepared powder was calcined at 700, 800, and 900 °C for 3 h under a nitrogen atmosphere with a heating rate of 3 °C min^−1^, followed by etching in 50 mL of 2 M HCl solution at 60 °C for 24 h and repeated washing with deionized water to remove the NaCl template. Finally, the sample was dried at 80 °C for 24 h in a convection oven and the product treated at 700, 800, and 900 °C was denoted as NC-Fe_AC1_, NC-Fe_AC2_, and NC-Fe_AC3_, respectively.

#### Preparations of the NC

5.0 g of NaCl and 0.5 g of dopamine hydrochloride were dissolved in 100 mL of deionized water, and the resulting solution was stirred for 24 h to form a homogeneous mixture. The above solution subsequently rapidly frozen in liquid nitrogen and freeze-dried at − 60 °C and 0.1 Pa for 3 days. The obtained powder was then calcined at 800 °C for 3 h under a nitrogen atmosphere with a heating rate of 3 °C min^−1^, followed by repeated washing with deionized water until the NaCl template was completely removed. Finally, the sample was dried at 80 °C for 24 h in a convection oven and the product was denoted as NC.

#### Preparations of the NC-Fe_NP_

5.0 g of NaCl, 0.5 g of dopamine hydrochloride, and 0.2 g of FeCl_2_‧4H_2_O were dissolved in 100 mL of deionized water and stirred for 24 h until a uniform solution was obtained. The mixture was then rapidly frozen using liquid nitrogen and freeze-dried at − 60 °C and 0.1 Pa for 3 days. The resulting powder was calcined at 900 °C for 3 h under a nitrogen atmosphere with a heating rate of 3 °C min^−1^, after which it was repeatedly washing with deionized water to remove the NaCl template. Finally, the sample was dried at 80 °C for 24 h in a convection oven and the obtained product was denoted as NC-Fe_NP_.

### Characterization

The morphology, microstructure, and elemental distribution of the samples were investigated by scanning electron microscopy (SEM, MERLIN Compact, Zeiss) and transmission electron microscopy (TEM, FEI Talos F200x, Thermo Fisher). Atomic distribution and coordination environments were further analyzed by double spherical aberration-corrected transmission electron microscope (double-Cs TEM, JEM-ARM300F, JEOL). The phase composition was determined by X-ray diffraction (XRD, Cu K*α*, *λ* = 1.5418 Å) with a scanning speed of 10° min^−1^ in the range of 3°–90°. Surface elemental valence states were analyzed by X-ray photoelectron spectroscopy (XPS, ESCALAB Xi+, Thermo Fisher) with Al K*α* X-ray source. X-ray absorption near-edge structure (XANES) and extended X-ray absorption fine structure (EXAFS) spectra at the Fe K-edge were recorded in fluorescence mode. Raman spectroscopy (Renishaw, In Via, *λ* = 523 nm) was used to probe structural features and defect states of the samples. Chemical bonds and functional groups of the samples were analyzed by Fourier-transform infrared spectroscopy (FT-IR, TENSOR II, Bruker). The thermal conductivity of the samples was measured using a thermal constants analyzer (Hot Disk TPS2500S). To evaluate electromagnetic properties, coaxial ring (outer diameter 7.00 mm, inner diameter 3.04 mm) was fabricated by dispersing 6 wt% of the samples into paraffin. The complex permittivity and permeability were measured multiple times in the range of 2–18 GHz using a vector network analyzer (E8363B, Agilent).

## Results and Discussion

### Design Principle of NC-Fe_ACX_ and Structural Characterizations

**Fig. 1:**
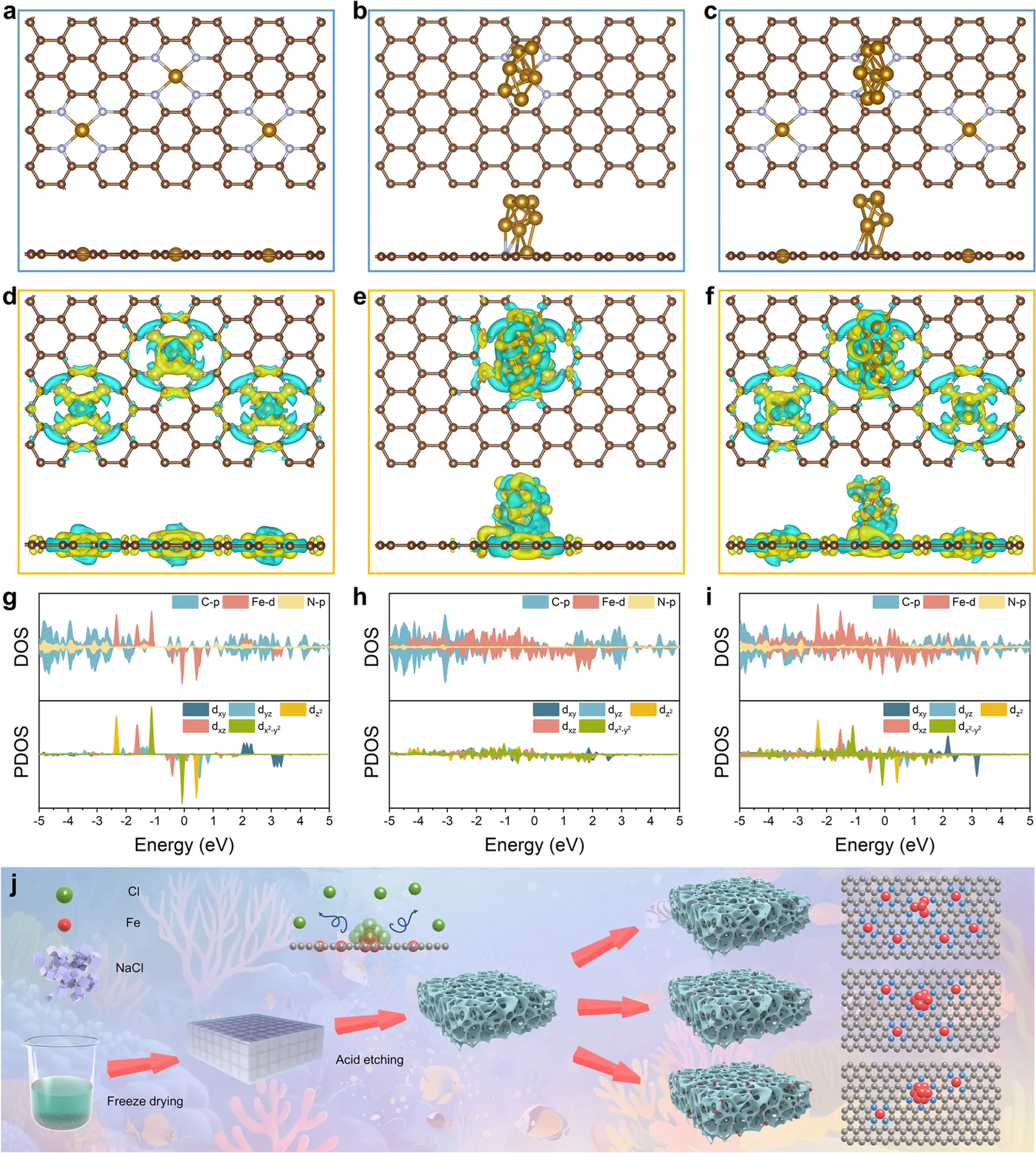
Model of **a** Fe_SA_, **b** Fe_AC_, and **c** Fe_AC-SA_ material. Charge density difference of **d** Fe_SA_, **e** Fe_AC_, and **f** Fe_AC-SA_ material. (The charge accumulation and depletion regions are represented by yellow and cyan, respectively.) Projected density of state (PDOS) and DOS of Fe *d* band in **g** Fe_SA_, **h** Fe_AC_, and **i** Fe_AC-SA_ material. **j** Schematic illustration of the synthesis process of NC-Fe_ACX_

To elucidate the intrinsic mechanism by which long‐range electron migration between Fe_AC_ and Fe_SA_ enhances EMW absorption, first-principles calculations were performed to evaluate the electronic structure and spatial charge distribution of three models: isolated Fe single atoms (Fe_SA_), coexisting Fe single atoms and clusters (Fe_SA-AC_), and Fe clusters (Fe_AC_) (Figs. 1a–c). In the Fe_SA_ model (Fig. 1d), the charge density difference analysis reveals a continuous charge accumulation zone along the Fe–N bond, accompanied by the delocalization of electrons from the bonding region to the periphery, forming a spatially symmetrical electron transfer feature. This phenomenon stems from the dominant *σ*-*π* conjugation hybridization between the pyridine nitrogen’s *p*_*z*_ orbital and Fe’s *d*_*xz*_/*d*_*yz*_ orbitals, and the effective overlap of *p—d* orbitals not only promotes the localized accumulation of electrons in the bonding region but also drives the symmetrical diffusion of electrons into the space outside the bond due to the delocalization characteristic of the conjugated system. Consistent with this, the projected density of states (PDOS) shows narrow and discrete *d* orbital peaks near the Fermi level, with a significant downward shift of the d orbital center (*ε*_*d*_) and a low density of states at the Fermi level (Fig. 1g), indicating that in the Fe-N_4_ coordination environment, *d* orbital electrons exhibit a clear localized feature due to the localization of orbital hybridization, and the range of electron delocalization is limited. In contrast, in the Fe_AC_ model (Fig. 1e), Fe–Fe atoms form bonding through strong *d—d* orbital overlap, with significant orbital wave function overlap leading to an expanded range of electron delocalization within the Fe aggregate; the charge redistribution is mainly concentrated within the Fe cluster and at the interface between the cluster and the carrier, reflecting a weakened orbital hybridization between the aggregate and the carrier, and the electron delocalization path is more confined within the metal cluster. Correspondingly, the *d* orbital broadening is the greatest, with a significant density of states and continuous *d* orbital occupation at the Fermi level (Fig. 1h), demonstrating typical metallic electron delocalization behavior, and electrons have high transportability [31]. Notably, in the Fe_SA-AC_ model (Fig. 1f), there is a cooperative coupling effect between isolated Fe single-atom sites and Fe clusters: The *π*-conjugated carbon carrier and the coordinating N atoms connecting the two form a continuous orbital conjugation network, creating a long-range polarization channel that mediates the long-range charge redistribution process involving the substrate. Driven by the local potential gradient and the mismatch of density of states, the d electrons of the Fe cluster preferentially inject into the conjugated system of the carbon carrier through orbital overlap and then migrate to the Fe single-atom sites via the *p* orbitals of the N atoms. The PDOS results (Fig. 1i) further confirm that in this model, the *d* orbital bandwidth is moderately broadened, *ε*_*d*_ is shifted upward, and the contribution of *d* orbital density of states near the Fermi level (especially the *d*_*xz*_/*d*_*yz*_ orbitals) is significantly enhanced, indicating an expanded range of electron delocalization, a reduced energy barrier for interorbital transitions, and a significantly improved electron transport capability. At the same time, the Fe cluster acts as an electron buffer center, achieving long-range electron transfer and multi-site cooperative migration through dynamic regulation of interface charges [32–34]. Therefore, constructing a composite system where single atoms and clusters coexist can significantly enhance electron mobility and control dielectric response by optimizing electron delocalization paths and orbital transition efficiency, providing a key guarantee for efficient attenuation of EM waves. Guided by simulation results, a series of NC-Fe_ACX_ absorbers with tunable Fe_AC_ sizes were rationally synthesized. As illustrated in Fig. 1j, dopamine hydrochloride and sodium chloride were dissolved in deionized water, followed by the introduction of ferrous chloride to form a homogeneous precursor solution. During freeze-drying, dopamine hydrochloride self-polymerized on the sodium chloride hard template (Fig. S1), where nitrogen atoms facilitated the uniform dispersion of Fe atoms. Subsequent pyrolysis under a nitrogen atmosphere, followed by acid leaching, produced N-doped absorbers containing homogeneously dispersed Fe_SA_ and Fe_AC_.

**Fig. 2:**
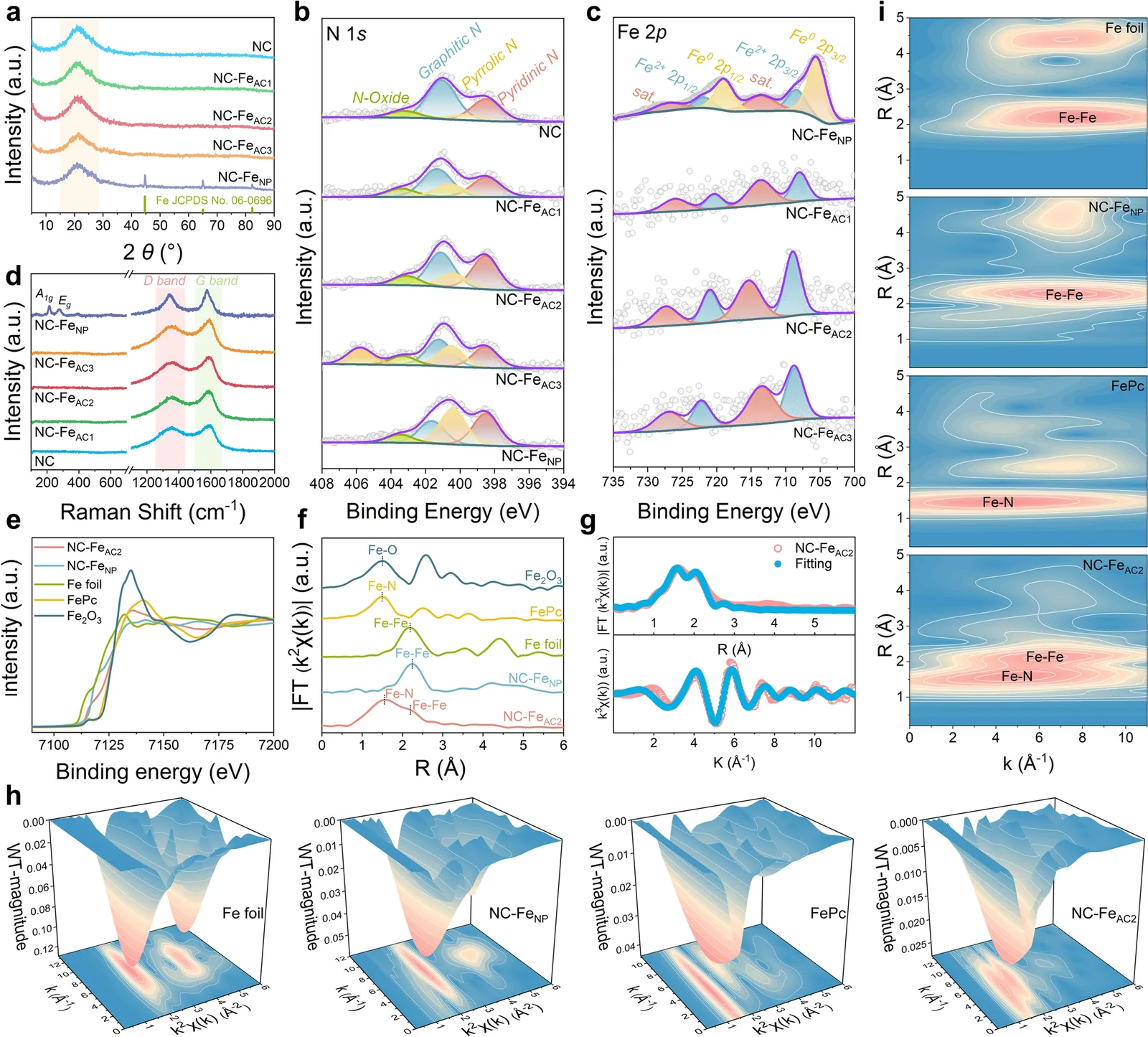
**a** XRD pattern of NC, NC-Fe_ACX_, and NC-Fe_NP_. **b** N 1*s*, **c** Fe 2*p* high-resolution XPS spectra, and **d** Raman spectra of NC, NC-Fe_ACX_, and NC-Fe_NP_. **e** Fe K-edge XANES spectra and **f** Fe K-edge k.^2^-weighted EXAFS spectra of NC-Fe_AC2_, NC-Fe_NP_, Fe foil, FePc, and Fe_2_O_3_. **g** Fitted Fe K-edge EXAFS curve of NC-Fe_AC2_ in the *k* and *R* space. **h** WT-EXAFS spectra and **i** projection of Fe foil, NC-Fe_NP_, FePc, and NC-Fe_AC2_

Figure 2a presents the X-ray diffraction (XRD) patterns of NC, NC-Fe_ACX_, and NC-Fe_NP_, which display two broad amorphous peaks at ~ 22° and ~ 44°, corresponding to the (002) and (101) planes of graphitic carbon. In addition, NC-Fe_NP_ exhibits the diffraction peaks at ~ 44.7°  ~ 65.1°, and ~ 82.3°, assigned to metallic Fe (JCPDS No. 06-0696) [35], indicating the aggregation of Fe nanoparticles in NC-Fe_NP_, whereas no such peak is observed in NC-Fe_ACX_. The surface chemical composition (Figs. S8-S10) and bonding configurations were further probed via X-ray photoelectron spectroscopy (XPS). The N 1*s* spectra (Fig. 2b) can be deconvoluted into pyridinic N (398.4 eV), pyrrolic N/M–N (399.8 eV), graphitic N (401.4 eV), and oxidized N (403.3 eV) [36]. Notably, the relative contribution of pyrrolic N/M–N increased significantly with the incorporation of Fe atoms, indicating that N species play a crucial role in anchoring Fe atoms. The Fe 2*p* spectrum (Fig. 2c) of NC-Fe_ACX_ shows Fe^2+^ 2*p*_3/2_ (710.3 eV) and Fe^2+^ 2*p*_1/2_ (723.4 eV) peaks with two satellite peaks. In contrast, NC-Fe_NP_ exhibits additional metallic Fe^0^ 2*p*_3/2_ (707.3 eV) and Fe^0^ 2*p*_1/2_ (720.4 eV) peaks, demonstrating that Fe atoms in NC-Fe_ACX_ exist exclusively as isolated Fe_SA_ and Fe_AC_ without aggregation into nanoparticles [37]. The FT-IR spectra exhibit characteristic bands at 3600–3000, 1250–1050, and 1650–1550 cm^‒1^, which are assigned to O–H/N–H, C–O–C, and C=C/C=N stretching vibrations in NC-Fe_ACX_ and NC-Fe_NP_, respectively. Notably, the functional groups-related bands gradually weaken from NC-Fe_AC1_ to NC-Fe_AC3_, indicating progressively enhanced deoxygenation/dehydrogenation upon more severe thermal treatment (Fig. S11). Raman spectra (Fig. 2d) reveal the characteristic D (~ 1350 cm^−1^) and G (~ 1580 cm^−1^) bands of carbon, corresponding to defect-induced vibrations and the ordered vibrations of sp^2^ carbons, respectively [38]. The *I*_*D*_*/I*_*G*_ ratios follow the order: NC (0.73) < NC-Fe_AC1_ (1.01) < NC-Fe_AC2_ (1.03) < NC-Fe_AC3_ (1.04) < NC-Fe_NP_ (1.10), indicating that the incorporation of Fe atoms introduces additional defects (Fig. S12). Specifically, Fe_AC_ generate coordination and boundary sites on the support that distort the local carbon structure, whereas larger nanoparticles exacerbate interfacial damage and local oxidation, thereby elevating the defect density. In addition to the D and G bands, the NC-Fe_NP_ sample exhibits distinct Fe–O Raman peaks at 225 cm^−1^ (A_1g_, symmetric stretching vibration of O along the Fe–O bond), 247, and 293 cm^−1^ (E_*g*_, bending and shear vibrations of O perpendicular to the Fe–O bond), which are attributed to the oxidation of Fe nanoparticles with high surface energy [39–41]. Extended X-ray absorption fine structure (EXAFS) and X-ray absorption near-edge structure (XANES) were performed to elucidate the fine structure and coordination environment of NC-Fe_AC2_ and NC-Fe_NP_. As shown in the Fe K-edge XANES spectra (Fig. 2e), the absorption edge of NC-Fe_AC2_ lies between Fe foil and Fe_2_O_3_, corresponding to an oxidation state between 0 and + 3. Moreover, NC-Fe_NP_ exhibits an edge position close to Fe foil, consistent with a near-zero valence state. Notably, the absorption edge of NC-Fe_AC2_ shows a slight shift relative to FePc (~ 7115 eV), implying that the introduction of Fe_AC_ induces distortions in the coordination structure of Fe_SA_ sites (Fe-N_4_) [36, 42]. The *k*^2^-weighted Fourier transform (FT) of the Fe K-edge EXAFS spectra (Fig. 2f) further reveals the local coordination environments of Fe atoms. NC-Fe_NP_ shows a dominant peak at ~ 2.2 Å, nearly identical to Fe foil, corresponding to the first-shell Fe–Fe scattering path and confirming the presence of Fe nanoparticles. In contrast, NC-Fe_AC2_ exhibits a main peak at ~ 1.5 Å, consistent with FePc and attributable to the first-shell Fe–N scattering path, while a secondary peak at ~ 2.2 Å reveals the coexistence of Fe–Fe bonds on the support [43, 44]. Quantitative EXAFS fitting in *k* and *R* space confirms the coexistence of isolated Fe-N_4_ sites and Fe_AC_ sites in the NC-Fe_AC2_ (Fig. 2g). Wavelet transform (WT) of EXAFS spectra further identifies the Metal-N and Metal–Metal coordination paths (Figs. 2h, i). FePc and Fe foil show a single maximum at ~ 4.0 and ~ 7.2 Å^−1^, assigned to Fe–N and Fe–Fe paths, respectively. In contrast, NC-Fe_AC2_ exhibits two distinct maxima at ~ 4.2 and ~ 6.4 Å^−1^, corresponding to Fe–N and Fe–Fe paths, significantly different from the WT signals of FePc and Fe foil [45, 46]. This divergence indicates that electron delocalization effect between Fe_SA_ sites and Fe_AC_ induces distortions in the local coordination environment, thereby facilitating polarization loss.

**Fig. 3:**
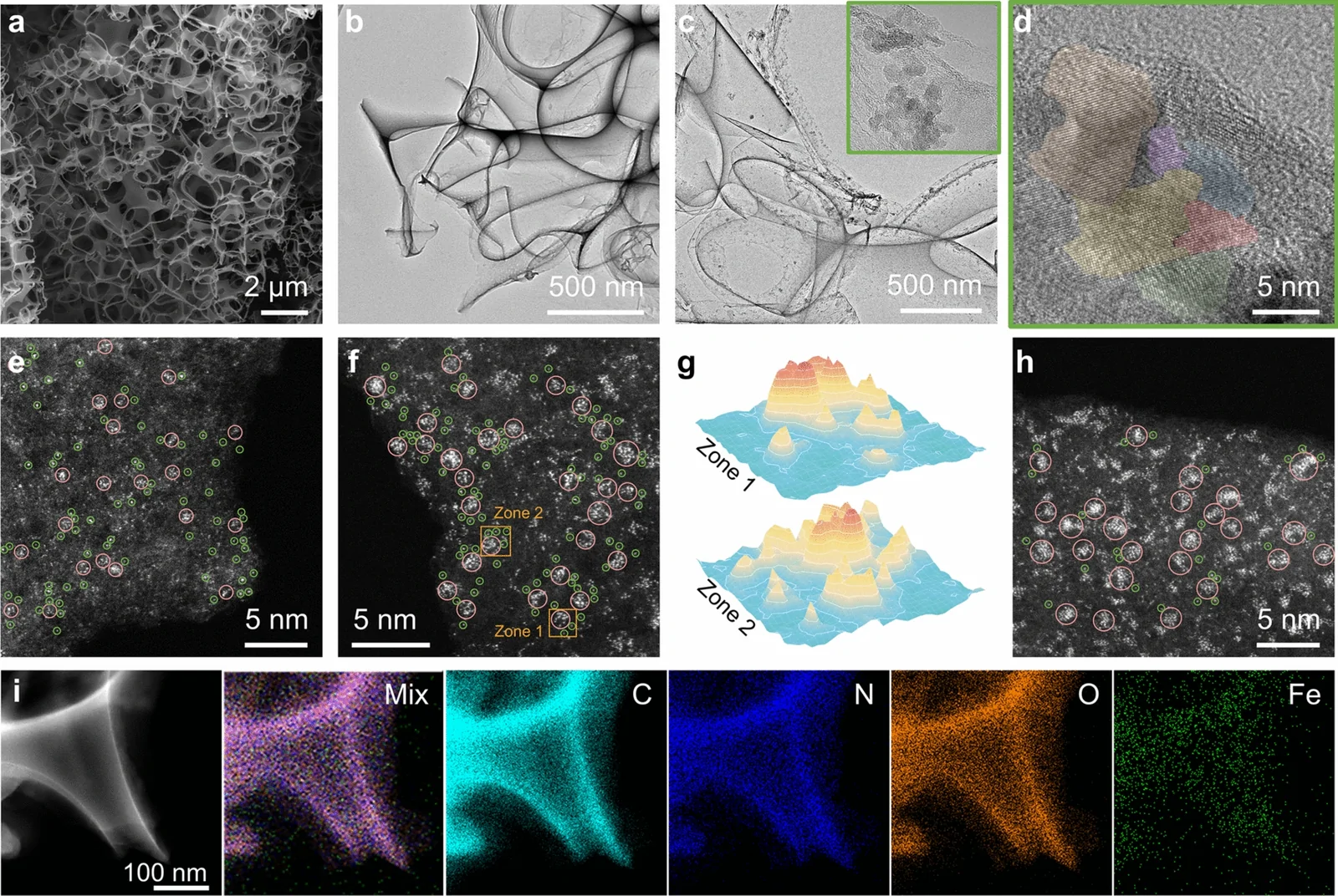
**a** SEM image of NC-Fe_AC2_. **b** TEM image of NC-Fe_AC2_. **c** TEM and **d** HRTEM image of NC-Fe_NP_. AC-HAADF-STEM image of **e** NC-Fe_AC1_, **f** NC-Fe_AC2_, and **h** NC-Fe_AC3_. **g** 3D fitting image of zones 1 and 2 in NC-Fe_AC2_. **i** EDX element mapping image of NC-Fe_AC2_

Scanning electron microscopy (SEM) and transmission electron microscopy (TEM) revealed that NC-Fe_ACX_ possessed a smooth three-dimensional (3D) porous coral-like morphology, indicating the homogeneous dispersion of Fe species (Figs. 3a–b and S2–S6). In contrast, the non-acid-treated NC-Fe_NP_ sample (Figs. 3c, d) exhibited extensive Fe nanoparticles aggregation with sizes below 10 nm induced by thermal migration of Fe atoms. To further elucidate the distribution states of Fe_SA_ and Fe_AC_ within the support, aberration-corrected high-angle annular dark-field scanning transmission electron microscopy (HAADF-STEM) was employed for atomic-scale analysis of NC-Fe_ACX_. As shown in Figs. 3e–h, Fe_AC_ surrounded by Fe_SA_ sites were uniformly distributed across the support. With increasing calcination temperature, the size of Fe_AC_ gradually increased, accompanied by a progressive decrease in the number of Fe_SA_ sites (Fig. S7). The 3D visualization fitting (Fig. 3g) vividly illustrated a cooperative island–archipelago configuration formed by Fe_SA_ and Fe_AC_. Furthermore, energy-dispersive X-ray (EDX) elements mapping confirmed the homogeneous distribution of C, N, O, and Fe in NC-Fe_ACX_ (Fig. 3i), with no Fe nanoparticles aggregation.

### Electromagnetic Performance and Mechanism

**Fig. 4:**
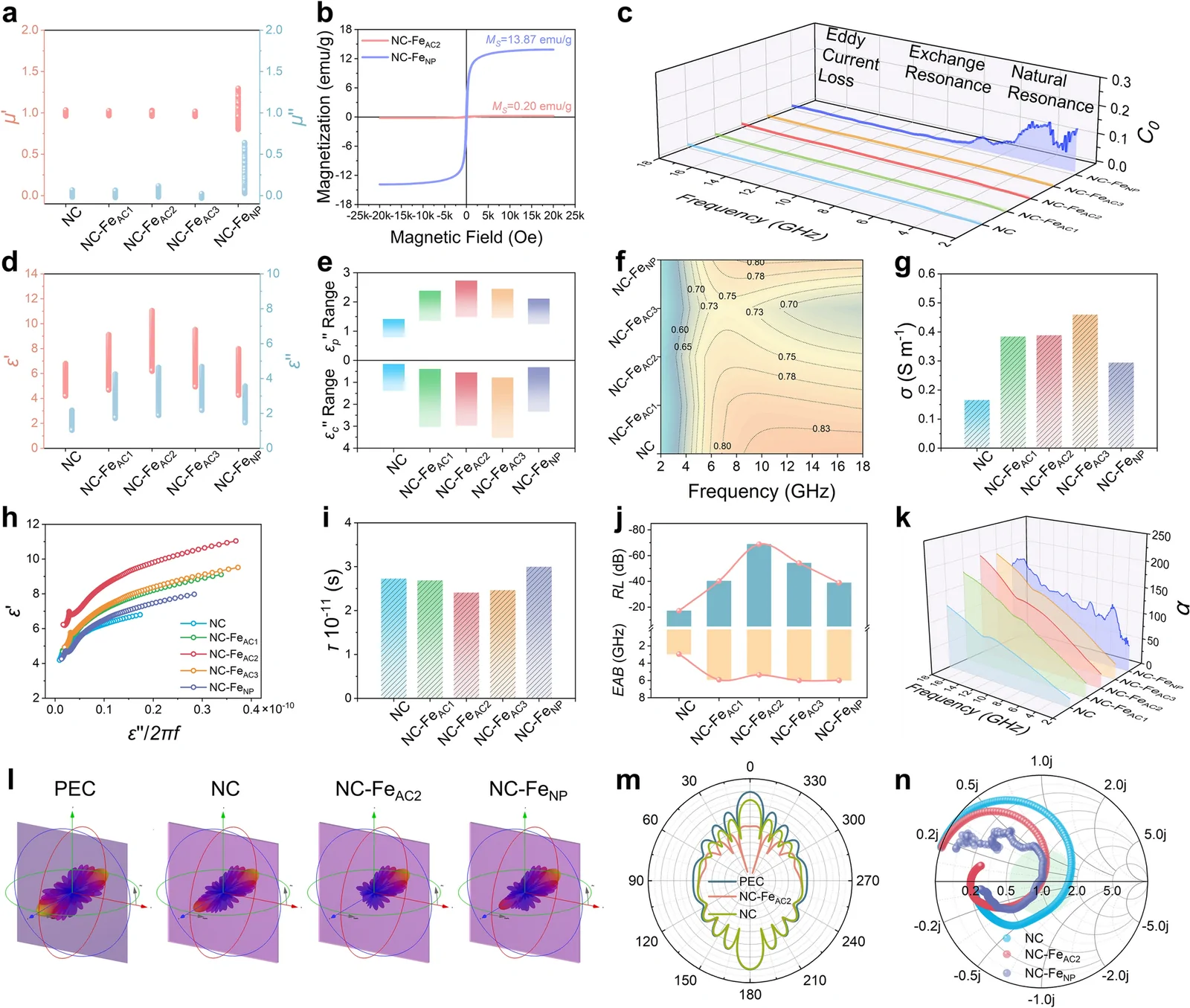
**a** Permeability ranges of NC, NC-Fe_ACX_, and NC-Fe_NP_. **b** Hysteresis loops of NC-Fe_AC2_ and NC-Fe_NP_. **c** The *C*_*0*_ values, **d** permittivity ranges, **e** *ε″*_*c*_ and *ε″*_*p*_ ranges, **f** contribution of polarization loss to dielectric loss, **g** *σ* values, **h** *ε′*-*ε″*/*2πf* curves, **i** *τ* values, **j** *RL* and *EAB* values, and **k** *α* values of NC, NC-Fe_ACX_, and NC-Fe_NP_. **l** 3D radar wave scattering signal of PEC, NC, NC-Fe_AC2_, and NC-Fe_NP_. **m** RCS simulation curves, and **n** smith chart of NC, NC-Fe_AC2_, and NC-Fe_NP_

To evaluate the EMW absorption performance, the intrinsic EM parameters—relative complex permittivity (*ε*_*r*_ = *ε′* − *jε″*) and relative complex permeability (*μ*_*r*_ = *μ′* − *jμ″*)—were measured at a filler loading of 6 wt%. As shown in Figs. 4a and S13, the *μ′* and *μ″* values of NC and NC-Fe_ACX_ are close to 1 and 0, respectively, indicating the absence of magnetism and confirming the uniform dispersion of Fe atoms consistent with the SEM and TEM observations. In contrast, the *μ′* and *μ″* values of NC-Fe_NP_ exceed 1 and 0, respectively, indicating its magnetic loss capability [47]. The magnetic hysteresis loops (Fig. 4b) further reveal the magnetic distinction of NC-Fe_AC2_ and NC-Fe_NP_. NC-Fe_AC2_ exhibits an almost linear hysteresis response through the origin, indicating negligible variation in magnetic induction (ΔB ≈ 0) under an external magnetic field, whereas NC-Fe_NP_ shows a smooth superparamagnetic “S”-shaped loop with nearly zero remanence and coercivity [48]. This behavior indicates that the Fe nanoparticles have a single-domain structure where magnetic moments randomly flip under thermal fluctuations, thereby eliminating remanence and coercivity at the macroscopic scale (Figs. 3c, d). Moreover, the saturation magnetization (*Ms*) of NC-Fe_NP_ (13.87 emu g^−1^) significantly exceeds that of NC-Fe_AC2_ (0.20 emu g^−1^), further corroborating its superior magnetic loss capability. The low-frequency resonance peaks of the *C*_*0*_ parameter are attributed to natural resonance and exchange resonance in the 2–18 GHz range, while the approach of *C*_*0*_ toward zero at higher frequencies suggests that eddy current loss occupies the dominant mechanism (Fig. 4c). The permittivity of the samples reflects the dielectric behavior under EMW. As shown in Figs. 4d and S14, the real (*ε′*) and imaginary (*ε″*) parts of permittivity represent energy storage and dissipation, respectively. All samples exhibit a frequency-dependent decrease in permittivity due to the intrinsic frequency dispersion effect. With the incorporation of Fe_SA_, Fe_AC_, and Fe_NP_, the *ε′* values of NC, NC-Fe_AC1_, NC-Fe_AC2_, NC-Fe_AC3_, and NC-Fe_NP_ oscillate within the ranges of 6.80–4.19, 9.11–4.68, 11.04–6.19, 9.52–4.93, and 7.97–4.26, respectively, while the *ε″* values fluctuate within 2.18–1.03, 4.25–1.71, 4.63–1.88, 4.69–2.17, and 3.56–1.47, respectively. Compared with NC, both NC-Fe_ACX_ and NC-Fe_NP_ exhibit higher *ε′* and *ε″* values, indicative of enhanced dielectric properties, as further corroborated by the dielectric loss tangent (tan *δ*_*ε*_). To better distinguish the contributions of conduction loss (*ε″*_*c*_) and polarization loss (*ε″*_*p*_), the permittivity was quantitatively fitted using the least-squares method (Figs. 4e, S15, and S16) [49]. The *ε″*_*c*_ values of NC-Fe_ACX_ are significantly higher than those of NC (1.40—0.16), consistent with the conductivity trend (Fig. 4g): NC-Fe_AC3_ (0.4597 S m^−1^) > NC-Fe_AC2_ (0.3886 S m^−1^) > NC-Fe_AC1_ (0.3843 S m^−1^) > NC-Fe_NP_ (0.2945 S m^−1^) > NC (0.1657 S m^−1^). These results demonstrate that Fe_AC_ and Fe_SA_ sites markedly enhance the electrical conductivity of the samples. This improvement originates from the electron delocalization space generated between Fe_AC_ and Fe_SA_, which promotes electron hopping in the support, optimizes the conductive network of NC-Fe_ACX_, and thereby strengthens conduction loss. Meanwhile, *ε"*_*p*_ reveals a volcano-like trend with increasing Fe_AC_ size. In the Fe SA model, the pyridine N *p*_*z*_ orbitals form *σ-π* conjugated hybridization with the Fe *d*_*xz*_*/d*_*yz*_ orbitals, and the accumulation and symmetrical delocalization of charges along the Fe–N bond form a directional dipole moment; when Fe_AC_ is introduced, the cooperative effect of the cluster and single atom distorts the symmetrical charge distribution of the Fe-N_4_ environment, intensifying the dipole polarization strength of the Fe–N bond, thereby enhancing the polarization loss (Fig. 4f). However, further increasing the size of Fe_AC_ causes the space hindrance effect of the clusters to intensify, thereby occupying the anchoring sites of the Fe_SA_, resulting in a synchronous decrease in the regional density of Fe_AC_ and Fe_SA_ on the *π*-conjugated carbon carrier surface. The decrease in site density will directly disrupt the effective overlap between the 3*d* orbitals of Fe_SA_ and the d-bands of Fe_AC_, hindering the delocalized transmission of interface electrons, and causing the electron state density distribution near the Fermi level to become discrete, unable to form a continuous polarization charge enrichment area. This not only weakens the cooperative regulatory effect of Fe_AC_ and Fe_SA_ sites on dielectric polarization, but is also highly consistent with density dependence of the “cluster-single atom” cooperative polarization channel in the Fe_SA-AC_ model—that is the polarization relaxation efficiency is positively correlated with the interface electron coupling density of the monomer–cluster. In summary, the synergistic effect of Fe_SA_ and size-controllable Fe_AC_ achieves a dynamic balance between conductive loss and polarization loss: Fe_AC_ enhances electronic conduction through *d—d* orbital delocalization, while Fe_SA_ regulates directional polarization via *σ-π* hybridization. The orbital conjugation network formed by the two promotes long-range charge migration and multi-site cooperative response (Fig. S17). This synergistic regulation mechanism of electronic delocalization and orbital polarization provides a key guarantee for optimizing dielectric response and enhancing electromagnetic wave attenuation efficiency. According to the Debye theory, the *ε′*-*ε″* relationship of samples reveals multiple Cole–Cole semicircles accompanied by a linear tail, indicating that both conduction loss and multiple polarization losses contribute to the EMW absorption (Fig. S18). The relaxation time (*τ*) represents the characteristic timescale for a system to recover from non-equilibrium to equilibrium state, which quantifies the response of dipoles or charges to an alternating EM field and serves as a critical parameter of polarization loss capability. As shown in the *ε′*-*ε″*/2*πf* plots, the relaxation times follow the order (Figs. 4h, i): NC-Fe_NP_ (3.00 × 10^−11^ s) > NC (2.73 × 10^−11^ s) > NC-Fe_AC1_ (2.69 × 10^−11^ s) > NC-Fe_AC3_ (2.47 × 10^−11^ s) > NC-Fe_AC2_ (2.41 × 10^−11^ s). Notably, NC-Fe_AC2_ exhibits the shortest relaxation time, confirming its superior polarization loss capability by enabling the most efficient conversion of EM energy into heat. Moreover, this ultrashort relaxation time further demonstrates that the delocalized space formed between Fe_SA_ and Fe_AC_ efficiently promotes electron hopping. In the coexisting Fe_SA_-Fe_AC_ system, the Fe_SA_ *3d* orbitals hybridize with the carbon support *2p* *π* orbitals, while Fe_AC_ *3d* orbitals weakly couple with the carbon orbitals, generating multicenter orbital overlap between Fe_SA_ and Fe_AC_ via the carbon support. This orbital coupling network facilitates electron delocalization among Fe_SA_, Fe_AC_, and carbon, which significantly enhances both conduction and polarization loss. The EMW absorption performance was systematically assessed by reflection loss (RL, where RL <  − 10 dB corresponds to 90% EMW absorption) and effective absorption bandwidth (EAB). The pristine NC sample without Fe_SA_ or Fe_AC_ exhibited poor performance, with an RL_*min*_ of − 17.17 dB and an EAB of 2.96 GHz at a thickness of 6.0 mm. With the incorporation of Fe_SA_ and Fe_AC_, the absorption performance of NC-Fe_ACX_ was significantly enhanced. NC-Fe_AC1_ achieved an RL_*min*_ of − 40.27 dB at 6.0 mm and an EAB of 5.92 GHz at 2.5 mm, while NC-Fe_AC2_ demonstrated better performance, reaching an RL_*min*_ of − 68.78 dB at 3.8 mm and an EAB of 5.36 GHz at 2.1 mm. The performance enhancement can be ascribed to the size modulation of Fe_AC_. Within the Fe_SA_-Fe_AC_ system, appropriately sized Fe_AC_ provides an optimal balance between orbital hybridization and electron migration: Partially overlapping 3d orbitals generate quasi-continuous energy bands while retaining a high density of Fe_SA_ active sites. Such a configuration enables effective coupling of Fe_AC_ *3d* orbitals with both the carbon *2p* *π* orbitals and Fe_SA_ *3d* orbitals, thereby establishing a multicenter hybridization network. This structure promotes extensive electron delocalization and accelerates charge transfer among Fe_SA_, Fe_AC_, and the carbon support, thereby significantly enhancing both polarization and conduction loss. Notably, further growth of clusters slightly degraded the performance of NC-Fe_AC3_, which showed an RL_min_ of − 54.16 dB at 3.5 mm and an EAB of 6.00 GHz at 2.4 mm. In contrast, substitution with Fe nanoparticles yielded only limited improvement, which NC-Fe_NP_ displays an RL_min_ of − 38.84 dB at 3.9 mm and an EAB of 6.00 GHz at 2.7 mm (Figs. S19, 20). Considering both the EAB-RL_min_ relationship (Fig. 4j) and attenuation constant (*α*), NC-Fe_AC2_ is identified as the optimal absorber, delivering the most efficient overall EMW absorption performance (Fig. 4k). The EMW absorption performance of absorbers depends on both attenuation capability and impedance matching. When the input impedance (*Z*_in_) falls within the green circle of the Smith chart (Fig. 4n), the reflection coefficient remains in the optimal range, with the center point (*Z′* = 1 + *j*0) denoting perfect matching [50]. The upper and lower halves of the horizontal axis correspond to inductive and capacitive characteristics, and resonance occurs when the impedance curve intersects this axis. With increasing frequency, the impedance curves of NC, NC-Fe_AC2_, and NC-Fe_NP_ shift from inductive to capacitive behavior. Notably, the impedance curve of NC-Fe_AC2_ passes directly through the center point (*Z′* = 1 + *j*0) and occupies the largest number of points within the green circle, evidencing its superior impedance matching. When *Z*_in_ approaches the impedance of free space (0.8 <|*Z*_in_/*Z*_*0*_|< 1.2), EMW can readily penetrate the absorber with minimal reflection. Consistently, NC-Fe_AC2_ exhibits the broadest red region (Fig. S23), while the correlation curves of reflection loss and impedance matching at different thicknesses are concentrated around |*Z*_in_/*Z*_*0*_|= 1, further confirming NC-Fe_AC2_ with the most favorable impedance matching (Fig. S24). The radar cross section (RCS) of samples was simulated using CST software to evaluate their practical application potential. A perfect electric conductor (PEC) substrate was coated with NC, NC-Fe_ACX_, and NC-Fe_NP_. As shown in the 3D radar reflection signals (Fig. 4l), absorbers could significantly reduce the scattering intensity, with NC-Fe_AC2_ exhibiting the lowest scattering signal. Furthermore, the RCS attenuation curves under varying 2D incidence angles (Figs. 4m and S25) further confirm this trend: NC-Fe_AC2_ (27.05 dB) > NC-Fe_AC3_ (16.95 dB) > NC-Fe_AC1_ (14.37 dB) > NC-Fe_NP_ (10.38 dB) > NC (6.38 dB). Among them, NC-Fe_AC2_ exhibits a substantially lower RCS value than other samples, which underscores its potential for practical applications at a 6 wt% filler loading.

### Properties and Applications of Multifunctional Films

**Fig. 5:**
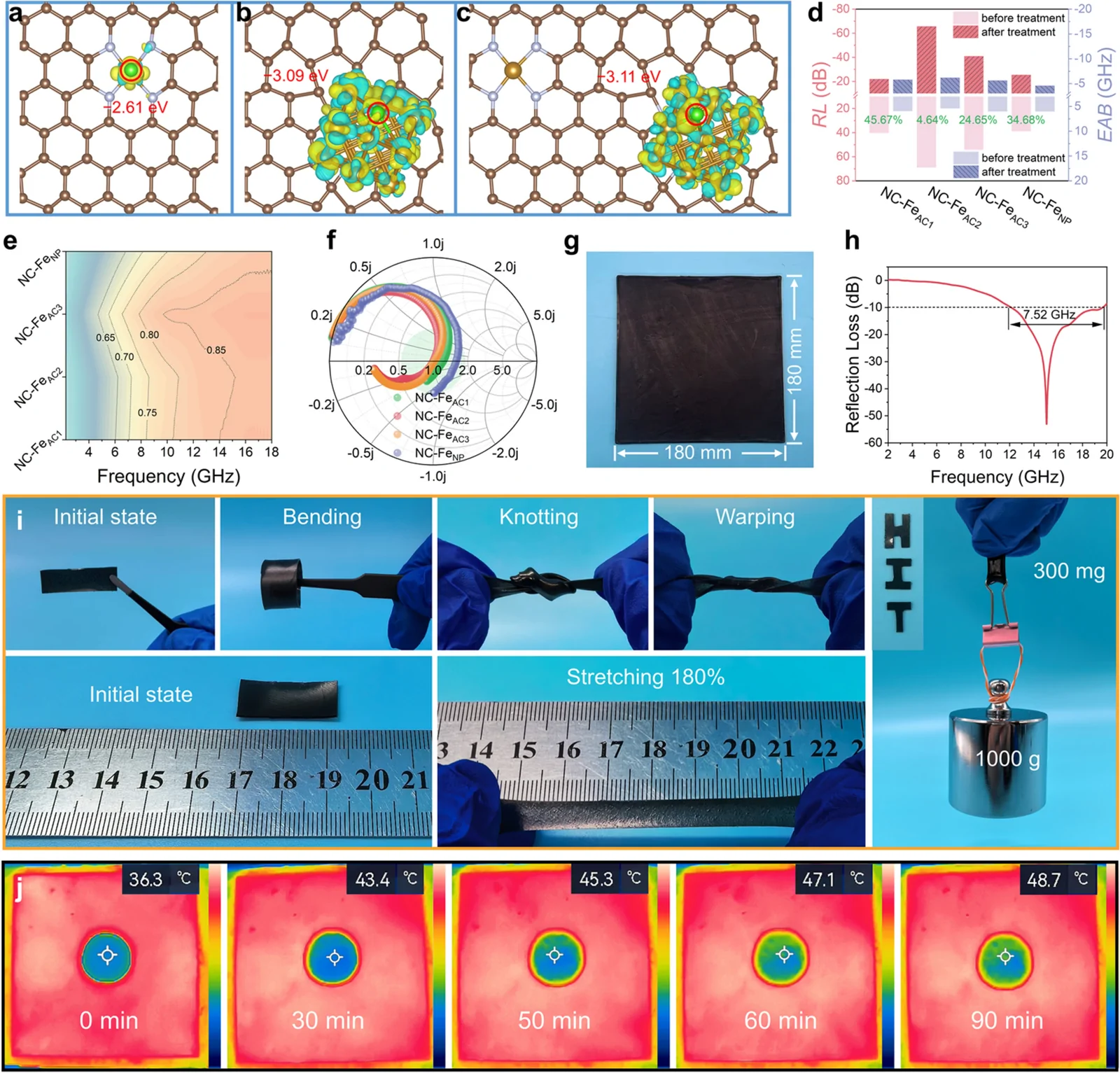
Difference charge density and Cl^‒^ adsorption energy of **a** Fe_SA_, **b** Fe_AC_, and **c** Fe_SA-AC_. **d** Comparison of *RL* and *EAB* values of NC-Fe_ACX_, and NC-Fe_NP_ before and after NaCl treatment (single measurement). **e** Contribution of polarization loss to dielectric loss, and **f** smith chart of NC-Fe_ACX_, and NC-Fe_NP_. **g** Image of NC-Fe_AC2_ film. **h** EMW performance of NC-Fe_AC2_ film measured by bow-method. **i** Images demonstrate the flexibility and stability of NC-Fe_AC2_ film. **j** Infrared images of NC-Fe_AC2_ film at 100 °C for 0–90 min

Charge density difference analysis and adsorption energy calculation reveal pronounced thermodynamic disparities in Cl^‒^ binding among the Fe species. As shown in Fig. 5a, Cl^−^ adsorption on the Fe_SA_ site gives an adsorption energy of − 2.61 eV, whereas a substantially stronger interaction is obtained on the Fe_AC_ site with a more negative value of − 3.09 eV (Fig. 5b). Notably, when Fe_SA_ coexists with Fe_AC_, the Cl^−^ adsorption energy on the Fe_AC_ domain further decreases to − 3.11 eV (Fig. 5c), evidencing a clear thermodynamic preference of Cl^−^ for Fe_AC_. Consistent with this prediction, the attenuation of EMW absorption performance in 3.5 wt% NaCl solution follows the order: NC-Fe_AC1_ (45.67%) > NC-Fe_NP_ (34.68%) > NC-Fe_AC3_ (24.65%) > NC-Fe_AC2_ (4.64%), indicating that the introduction of appropriately sized Fe_AC_ significantly enhances the resistance of absorbers against Cl^−^ corrosion (Figs. 5d and S33–S36). This improvement arises from the preferential accumulation of Cl^−^ on Fe_AC_, which generates a locally enriched negative charge region and thereby effectively reduces the concentration of free Cl^−^ near Fe_SA_, thus mitigating the poisoning effect and enhancing the absorption stability of absorbers in marine environments. The polarization capability is improved after NaCl treatment due to Cl^−^-induced local charge redistribution in Fe_AC_, which generates additional dipoles and promotes polarization loss (Figs. 5e, f and S27–S32). To validate the EMW absorption performance under practical conditions, bow-shaped method measurements of treated films (180 × 180 × 2 mm) revealed an *EAB* of 7.52 GHz and a RL_min_ of − 53.04 dB, consistent with simulation results (Figs. 5g, h and S37). Apart from excellent EMW absorption, practical applications in marine environments also require mechanical robustness, flexibility, and thermal insulation. NC-Fe_AC2_ films retained structural integrity under bending, knotting, and twisting, exhibiting excellent flexibility and mechanical stability (Fig. 5i). Moreover, NC-Fe_AC2_ films demonstrated remarkable ductility and elasticity, tolerating elongation up to 180%. A single 300 mg film could support a 1000 g load, underscoring the exceptional mechanical performance. To further evaluate the influence of mechanical deformation on the EMW absorption performance of the films, we compared its absorption behavior before and after tensile stretching. The results demonstrate that mechanical deformation exerts a negligible effect on the EMW absorption performance (Fig. S38). Thermal insulation performance was further evaluated for a 2 mm-thick NC-Fe_AC2_ aerogel film (Fig. 5j), which exhibits a low thermal conductivity of 0.077 W M^−1^ k^−1^. The infrared thermal imaging results showed that, when placed on a 100 °C heating plate for 90 min, the surface temperature increased by only 12.4 °C (Fig. S39), indicating excellent thermal insulation capability. Overall, NC-Fe_AC2_ films integrate efficient EMW absorption (Fig. S40), remarkable mechanical stability, and excellent thermal insulation performance, underscoring the potential for applications in naval vessels and marine equipment, including radar stealth and device protection [50–55].

## Conclusions

In summary, we developed a Fe cluster–single-atom synergistic absorber (NC-Fe_AC2_) for high-efficiency electromagnetic wave attenuation under marine environments. At a low loading of 6 wt%, NC-Fe_AC2_ delivers a minimum reflection loss of − 68.78 dB and an effective absorption bandwidth of ~ 6 GHz. Fe clusters (Fe_AC_) and Fe single atoms (Fe_SA_) anchored on a *π*-conjugated carbon support create long-range charge delocalization and multicenter coupling, which strengthen conduction loss. Concurrently, size-tunable Fe_AC_ subtly disrupts the local symmetry of Fe-N_4_ coordination sites, inducing reconfiguration of Fe *3d*-N *p* orbital hybridization and asymmetric electron density distribution near the Fermi level. Not only enhances dipole polarization relaxation efficiency, but also optimizes impedance matching via dielectric constant regulation. Thermodynamically, preferred Cl^−^ adsorption on Fe_AC_ creates locally negative regions that electrostatically shield and stabilize Fe_SA_ sites, suppressing chloride-induced poisoning and preserving dielectric response in marine environments. The NC-Fe_AC2_ film also exhibits straightforward processability, mechanical flexibility, and thermal insulation, highlighting its practicality for multifunctional maritime EM protection and illustrating a generalizable strategy that couples clusters with single atoms to co-optimize performance and durability.

## Supplementary Information

Below is the link to the electronic supplementary material.

Supplementary file1 (DOCX 22344 KB)
