# Supplementary material for: Electronic Delocalization of Fe Atom–Cluster for Long-Term Stable Electromagnetic Wave Absorption in Marine Environments
Source: Nanomicro Lett. 2026 May 11;18:364. doi: 10.1007/s40820-026-02210-y (PMC13161427; doi:10.1007/s40820-026-02210-y)
Supplement: Supplementary file 1 — Supplementary file1 (DOCX 22344 KB) [file 40820_2026_2210_MOESM1_ESM.docx]

Supporting Information for

**Electronic Delocalization of Fe Atom-Cluster for Long-Term Stable Electromagnetic Wave Absorption in Marine Environments**

Shaocong Zhong^1^, Xinyu Wang^1^, Rurong Zou^1, 2^, Chang Long^2^, Pianpian Zhang^1,^ *, Xueting Zhang^1^, Zihao Zhao^1^, Ying Liu^1^, Can Cui^1^, Yanan Yang^1,^ * and Long Xia^1,^ *

^1^ College of Materials Science and Engineering, Harbin Institute of Technology (Weihai), Weihai 264209, P. R. China

^2^ Aerospace Science and Industry Wuhan Magnetism-Electron Co., Ltd, Wuhan 430074, P. R. China

* Corresponding authors. E-mail: [zhangpianpian1993@126.com](mailto:zhangpianpian1993@126.com) (Pianpian Zhang); [yangyanan310@163.com](mailto:yangyanan310@163.com) (Yanan Yang); [xialonghit@gmail.com](mailto:xialonghit@gmail.com) (Long Xia)

**S1 Supplementary Text**

***S1.1 Materials***

Acrylamide (C_3_H_5_NO, AR), 2-Hydroxy-2-methylpropiophenone (C_10_H_12_O_2_, 97%), Polyethylene glycol diacrylate (PEGDA, C_5_H_10_O_4_, Mr~1000), and Carboxymethyl cellulose were both purchased from Shanghai Aladdin Biochemical Technology Co., Ltd (Shanghai, China). Soda-lime glass and PET sheet were purchased from Zhuhai Kaiwei Optoelectronic Technology Co., Ltd (Guangdong, China).

***S1.2 Preparations of the*** ***NC-Fe_AC2_ film***

10 g of acrylamide monomer was mixed with a 5 mg of crosslinker (polyethylene glycol diacrylate), and 8mg of photoinitiator (2-hydroxy-2-methylpropiophenone). The mixture was heated and stirred in a water bath at 80 °C until a clear solution was obtained. Square soda-lime glass plates (0.5 cm thick) and PET sheets (0.2 cm thick) were rinsed three times with deionized water and ethanol, respectively, and then dried for use as the bottom substrate and the top cover for ion-gel fabrication. The precursor solution was drop-cast onto the glass substrate, and glass spacers (2.0 mm thick) were used to define the gap before covering with the PET sheet. A UV lamp (365 nm) was positioned 2 cm above the precursor, followed by UV curing for 1 min. Finally, the top plate was removed and the gel film was peeled off.

***S1.3 Preparations of the NC-Fe_AC2_ aerogel film***

0.5 g of NC-Fe_AC2_ was added to 10 mL of deionized water and ultrasonicated for 30 min to obtain a homogeneous suspension (Solution A). Separately, 4.0 g of carboxymethyl cellulose was dissolved in 15 mL of deionized water and stirred continuously at 60 °C for 1 h to form a uniform solution (Solution B). Solution A was then slowly dropwise added to Solution B under continuous stirring, and the mixture was further stirred for 30 min to ensure uniform blending. The resulting mixture was transferred into a mold, rapidly frozen with liquid nitrogen, and subsequently freeze-dried at ‒60 °C under 0.1 Pa for 3 days to obtain the aerogel film.

***S1.4 Measurement of electromagnetic parameters***

The reflection loss of absorbers with various matching thicknesses in the 2−18 GHz range was calculated by complex permittivity (*ε*_r_) and complex permeability (*μ*_r_) based on transmission line theory. where *Z*_in_ is the input impedance of absorber, *Z*_0_ is the impedance of free space, *f* is the frequency of electromagnetic wave, *d* is the thickness of absorber, and *c* is the speed of light, respectively.

$\varepsilon_{r}=\varepsilon'-j\varepsilon''$ (S1)

$\mu_{r}=\mu'-j\mu''$ (S2)

$RL(\text{dB})=-20\lg\left|\frac{(Z_{in}-Z_{0})}{(Z_{in}+Z_{0})}\right|$ (S3)

$Z_{in}=Z_{0}\sqrt{\frac{\mu_{r}}{\varepsilon_{r}}}\tanh\left(\frac{2j\pi fd\sqrt{\mu_{r}\varepsilon_{r}}}{c}\right)$ (S4)

Based on Debye dipole relaxation theory, the model between *ε*′ and *ε*″ can be expressed by equation S5, and the complex permittivity imaginary part (*ε*″) can be separated into polarization loss (*ε*_p_″) and conduction loss (*ε*_c_″). where *ε*_s_ is static permittivity, *ε*_∞_ is relative permittivity, *ω* is angular frequency, *τ* is polarization relaxation time and *σ* is electrical conductivity, respectively.

$\left(\varepsilon'-\frac{\varepsilon_{s}+\varepsilon_{\infty}}{2}\right)^{2}+\left(\varepsilon''\right)^{2}=\left(\frac{\varepsilon_{s}+\varepsilon_{\infty}}{2}\right)^{2}$ (S5)

$\varepsilon''=\frac{\omega\tau\left(\varepsilon_{s}-\varepsilon_{\infty}\right)}{1+\omega^{2}\tau^{2}}+\frac{\sigma}{\omega\varepsilon_{0}}=\varepsilon''_{p}+\varepsilon''_{c}$ (S6)

The attenuation constant (*α*) and impedance matching (*Z*) can be calculated by the following equations.

$\alpha=\frac{\sqrt{2}\pi f}{c}\sqrt{\left(\mu''\varepsilon''-\mu'\varepsilon'\right)+\sqrt{\left(\mu'\varepsilon''+\mu''\varepsilon'\right)^{2}+\left(\mu''\varepsilon''-\mu'\varepsilon'\right)^{2}}}$ (S7)

$Z=\left|\frac{Z_{in}}{Z_{0}}\right|$ (S8)

The contribution of the dielectric loss and magnetic loss can be calculated by the following equations.

$\tan\delta_{\varepsilon}=\frac{\varepsilon''}{\varepsilon'}$ (S9)

$\tan\delta_{\mu}=\frac{\mu''}{\mu'}$ (S10)

***S1.5 Density functional theory (DFT) calculations***

All the DFT calculations were conducted based on the Vienna Ab initio Simulation Package (VASP). The exchange-correlation potential was described by the Perdew-Burke-Ernzerhof (PBE) generalized gradient approach (GGA). The electron-ion interactions were accounted by the projector augmented wave (PAW). All DFT calculations were performed with a cut-off energy of 400 eV, and the 3×3×1 Monkhorst-Pack grid k-points were selected to sample the Brillouin zone integration. The energy and force convergence criteria of the self-consistent iteration were set to 10^‒4^ eV and ‒0.02 eV Å^‒1^, respectively. DFT-D3 method is adopted to describe the van der Waals interaction.

**S2 Supporting Figures**


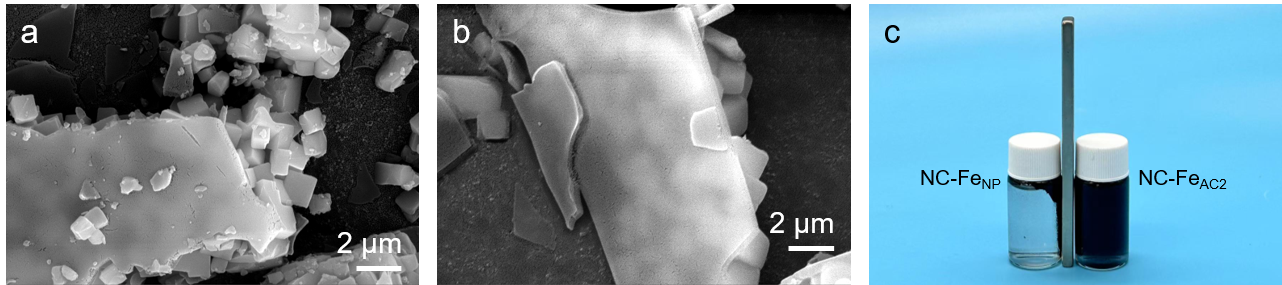


**Fig. S1** Characteristic of NC-Fe_ACX_ precursor structure: a, b) SEM image, c) Magnetic property images of NC-Fe_AC2_ and NC-Fe_NP_


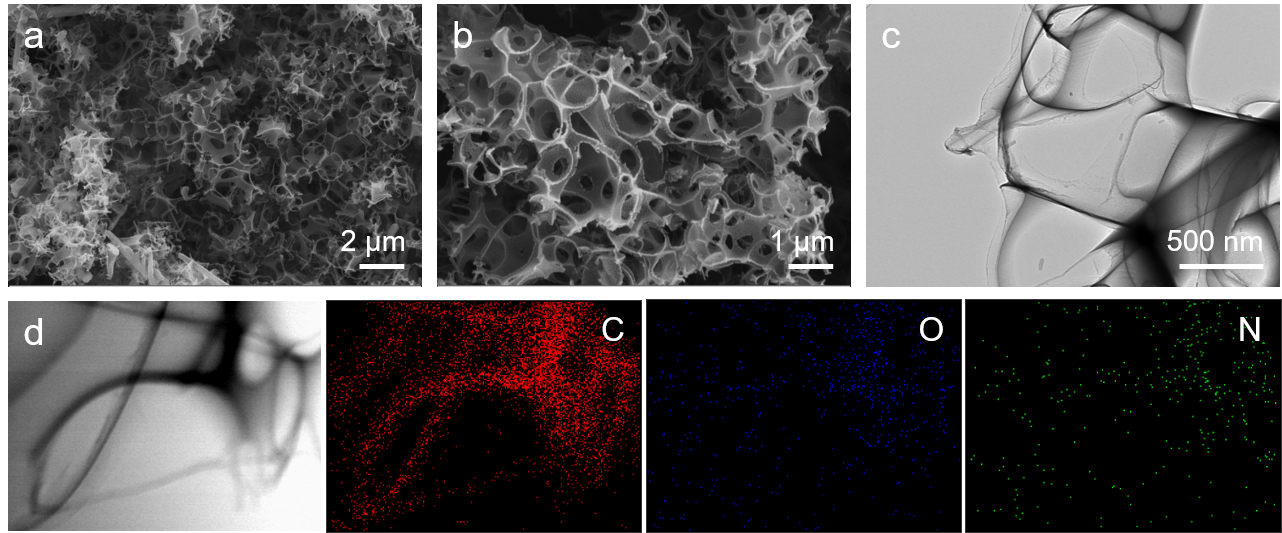


**Fig. S2** Characteristic of NC structure: a, b) SEM image, c) TEM image and d) EDX elements mapping image for C, O and N


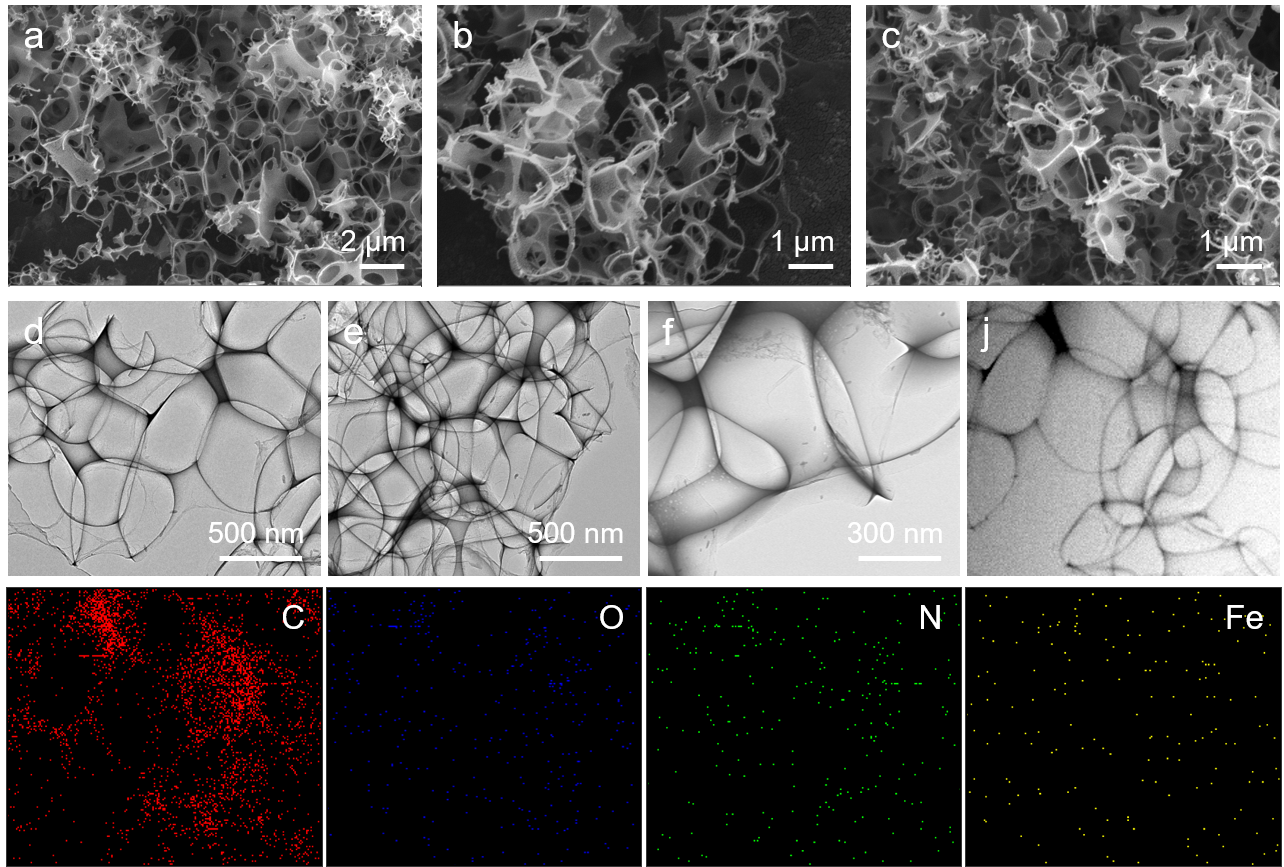


**Fig. S3** Characteristic of NC-Fe_AC1_ structure: a-c) SEM image, d-f) TEM image and j) EDX elements mapping image for C, O, N and Fe


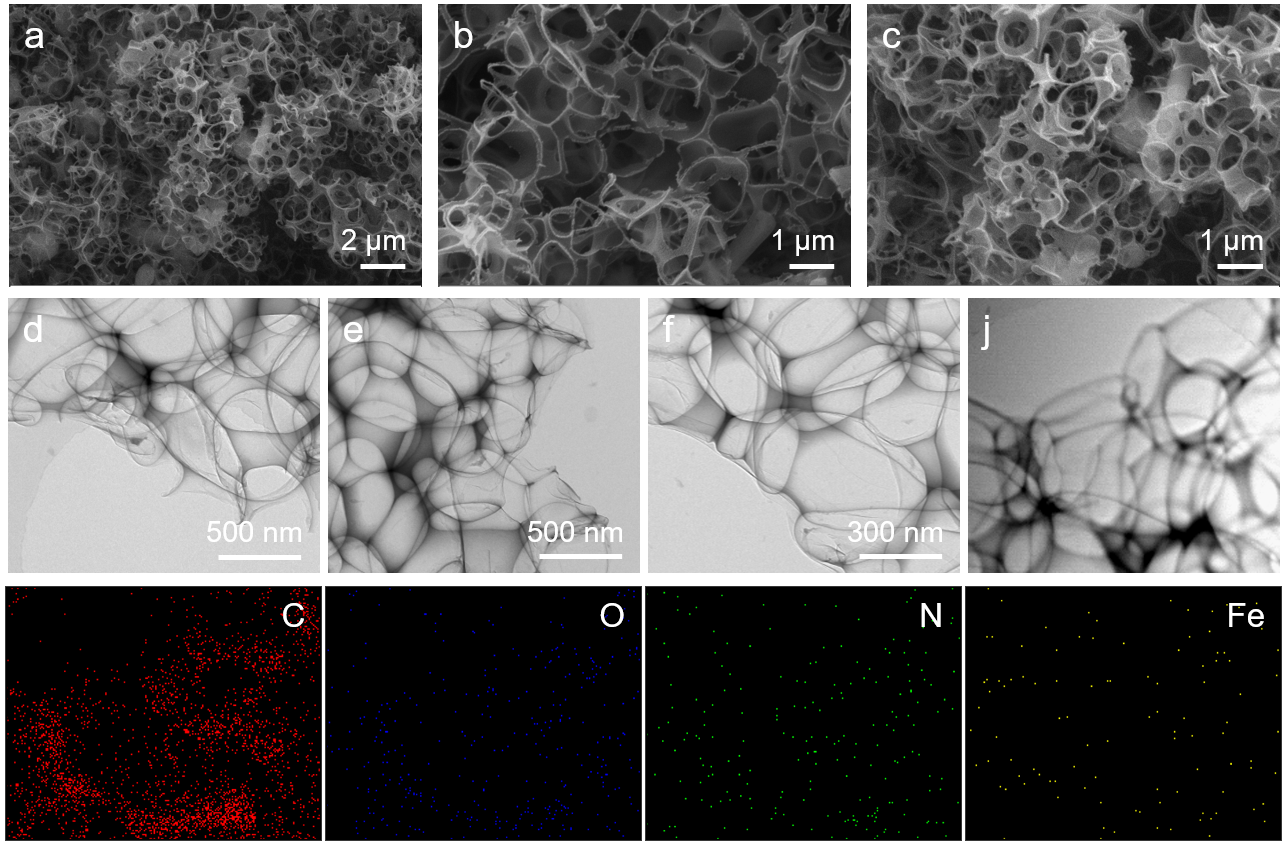


**Fig. S4** Characteristic of NC-Fe_AC2_ structure: a-c) SEM image, d-f) TEM image and j) EDX elements mapping image for C, O, N and Fe


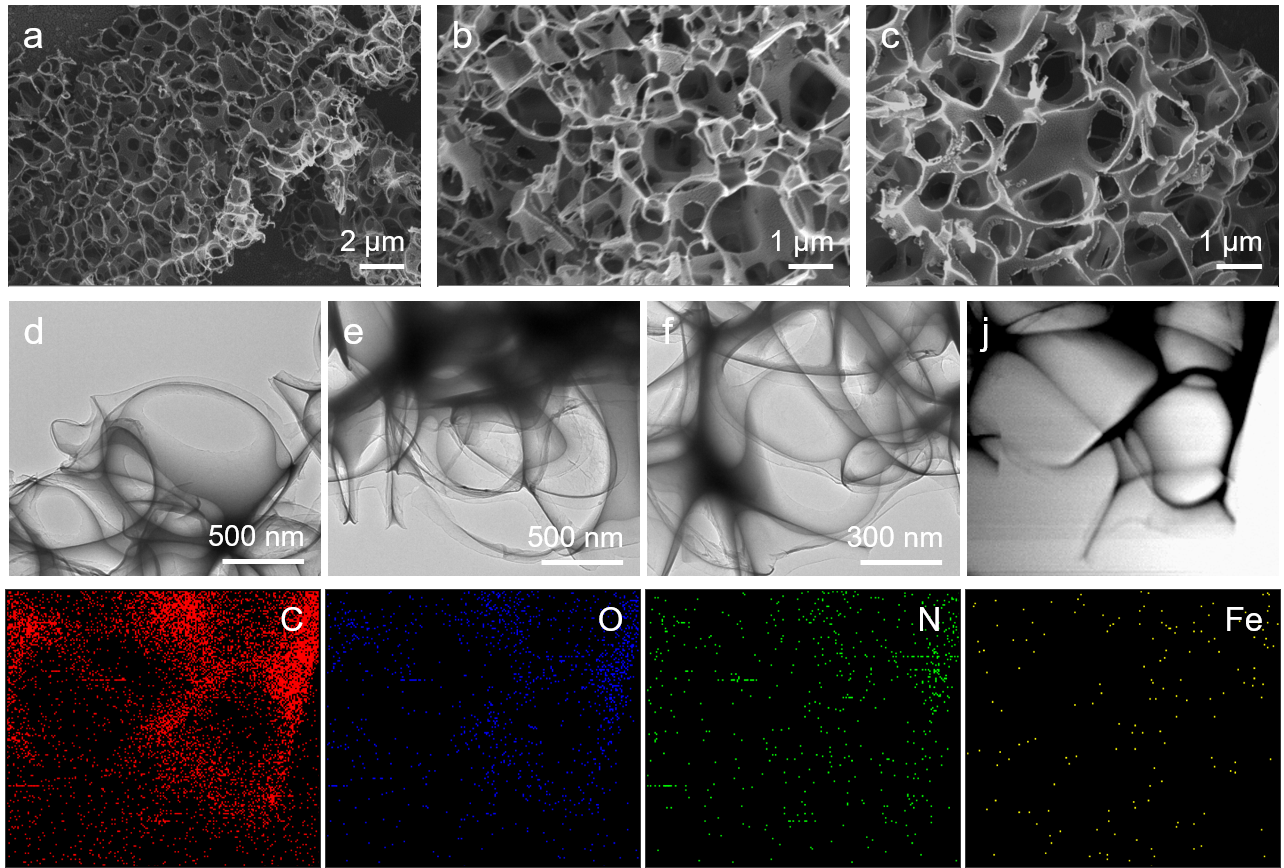


**Fig. S5** Characteristic of NC-Fe_AC3_ structure: a-c) SEM image, d-f) TEM image and j) EDX elements mapping image for C, O, N and Fe


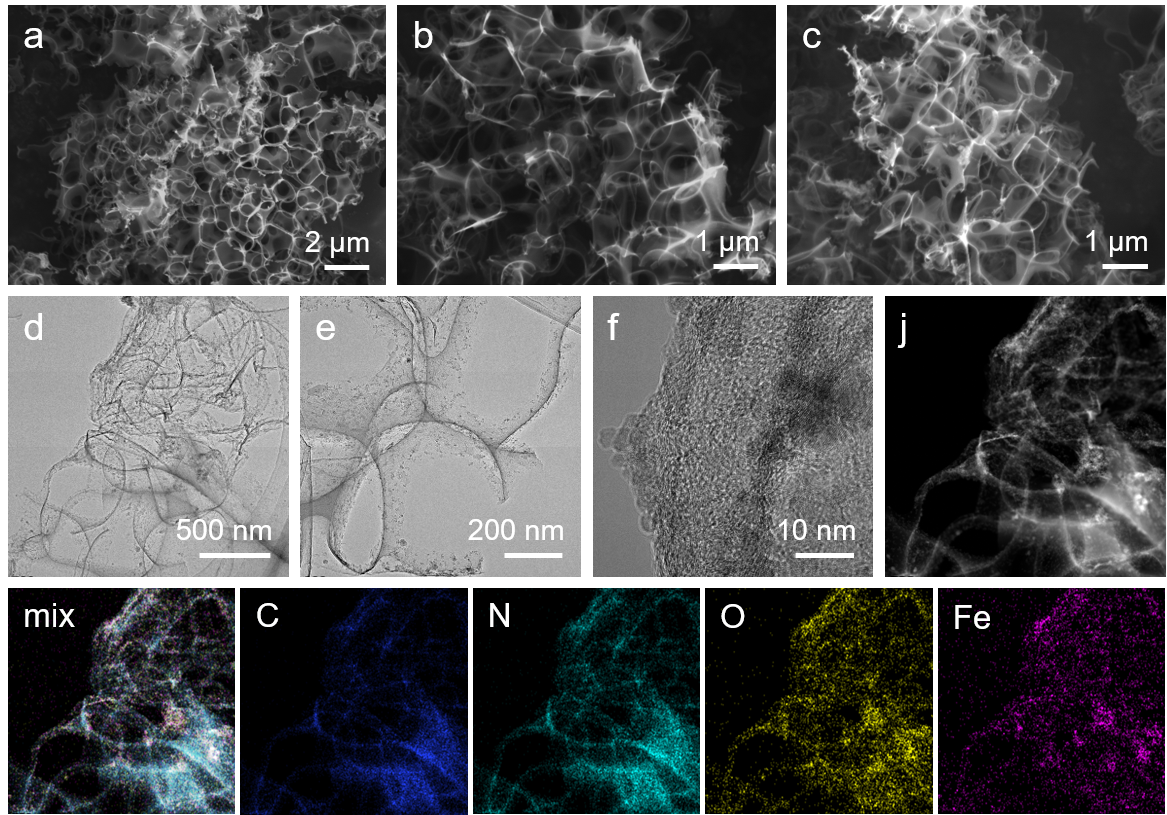


**Fig. S6** Characteristic of NC-Fe_NP_ structure: a-c) SEM image, d-f) TEM image and j) EDX elements mapping image for C, O, N and Fe


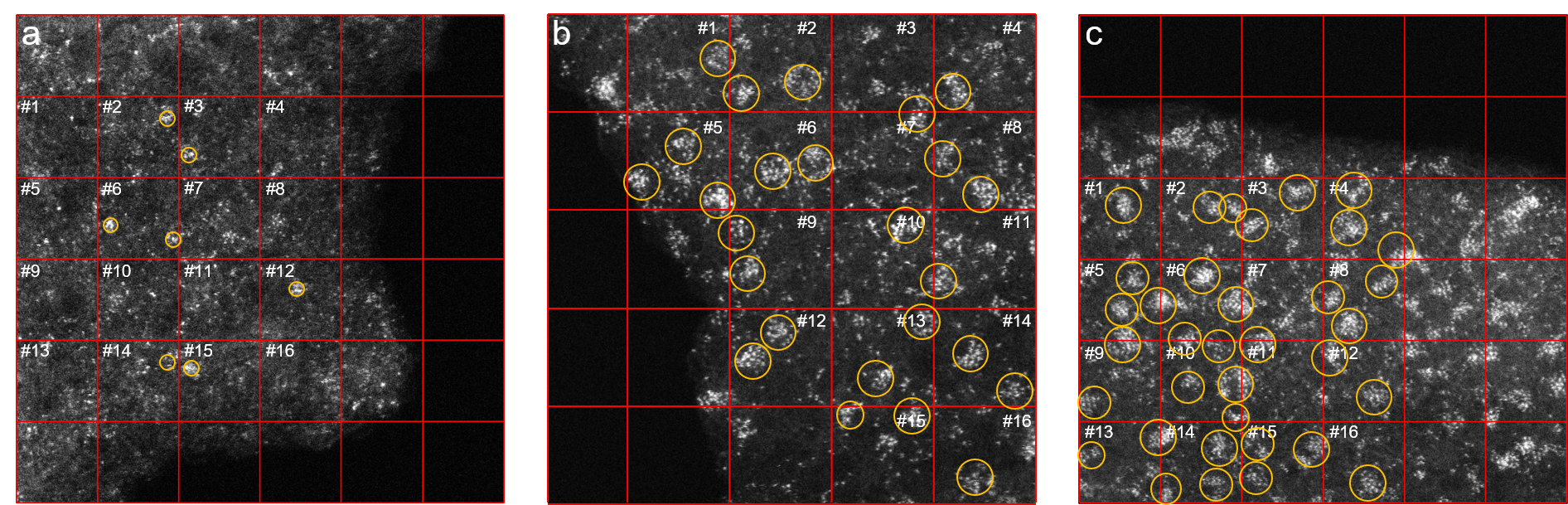


**Fig. S7** AC-HAADF-STEM image of a) NC-Fe_AC1_, b) NC-Fe_AC2_, and c) NC-Fe_AC3_ (The red squares are of the same size and yellow circle represents Fe_AC_)


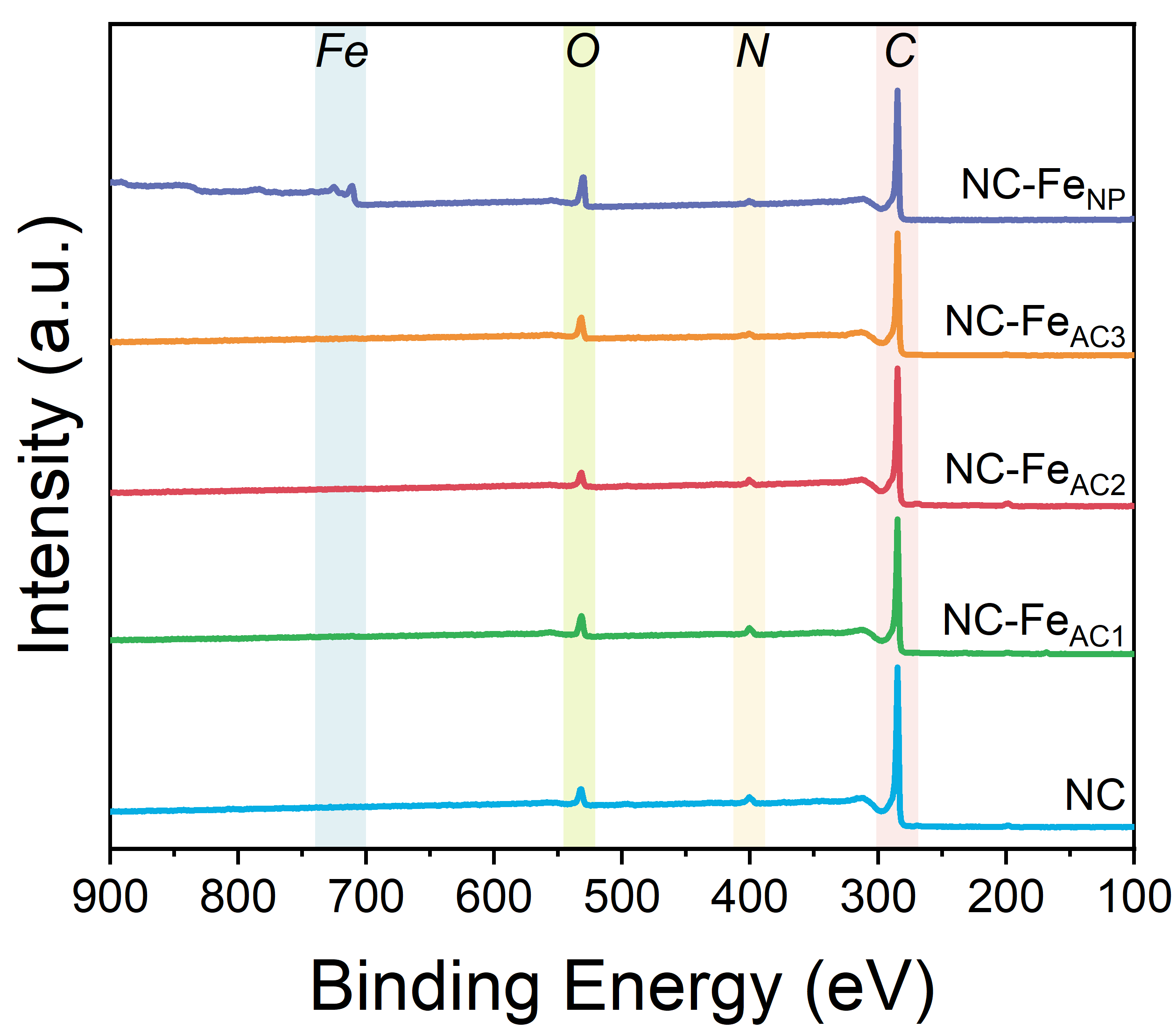


**Fig. S8** XPS survey spectra of NC, NC-Fe_ACX_, and NC-Fe_NP_


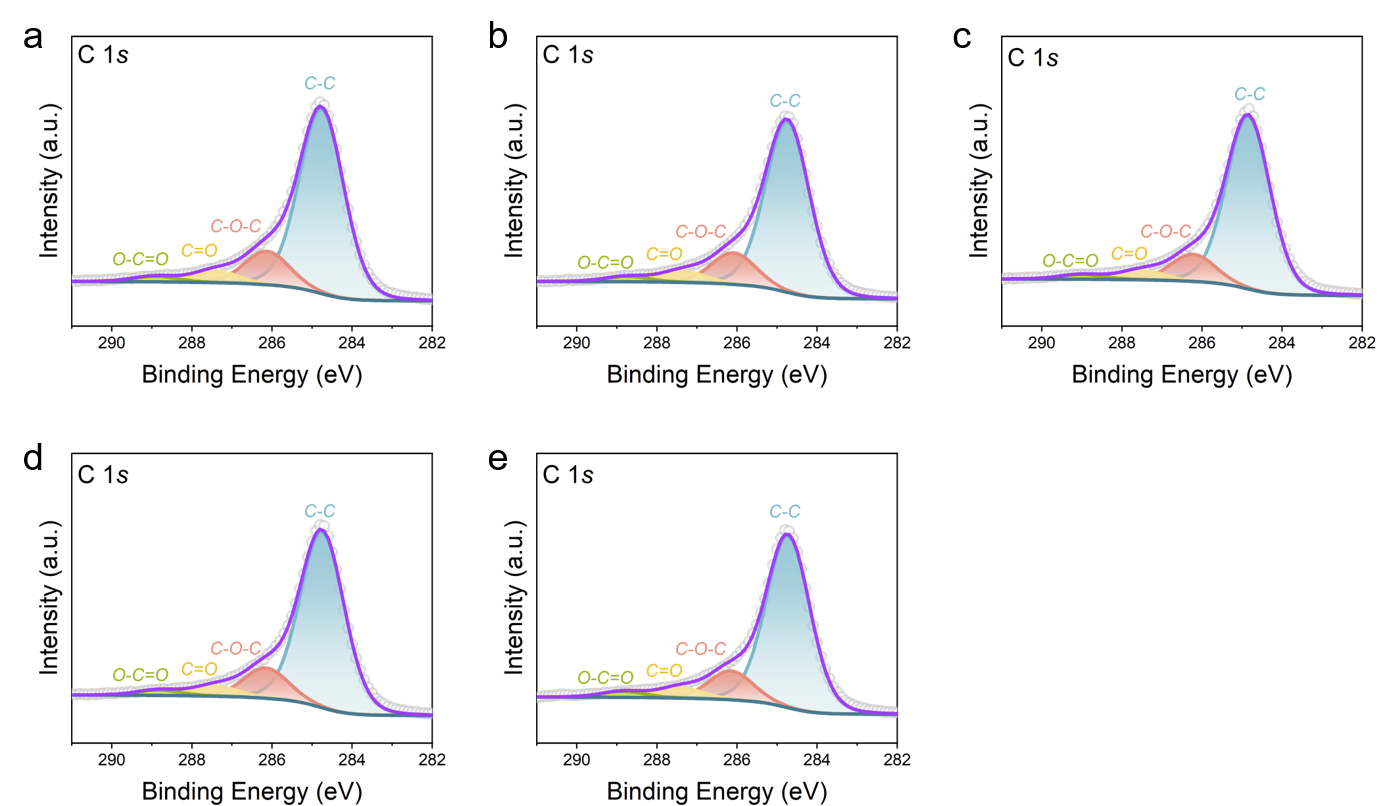


**Fig. S9** High-resolution XPS spectrum of C 1s spectra for a) NC, b) NC-Fe_AC1_, c) NC-Fe_AC2_, d) NC-Fe_AC3_, and e) NC-Fe_NP_


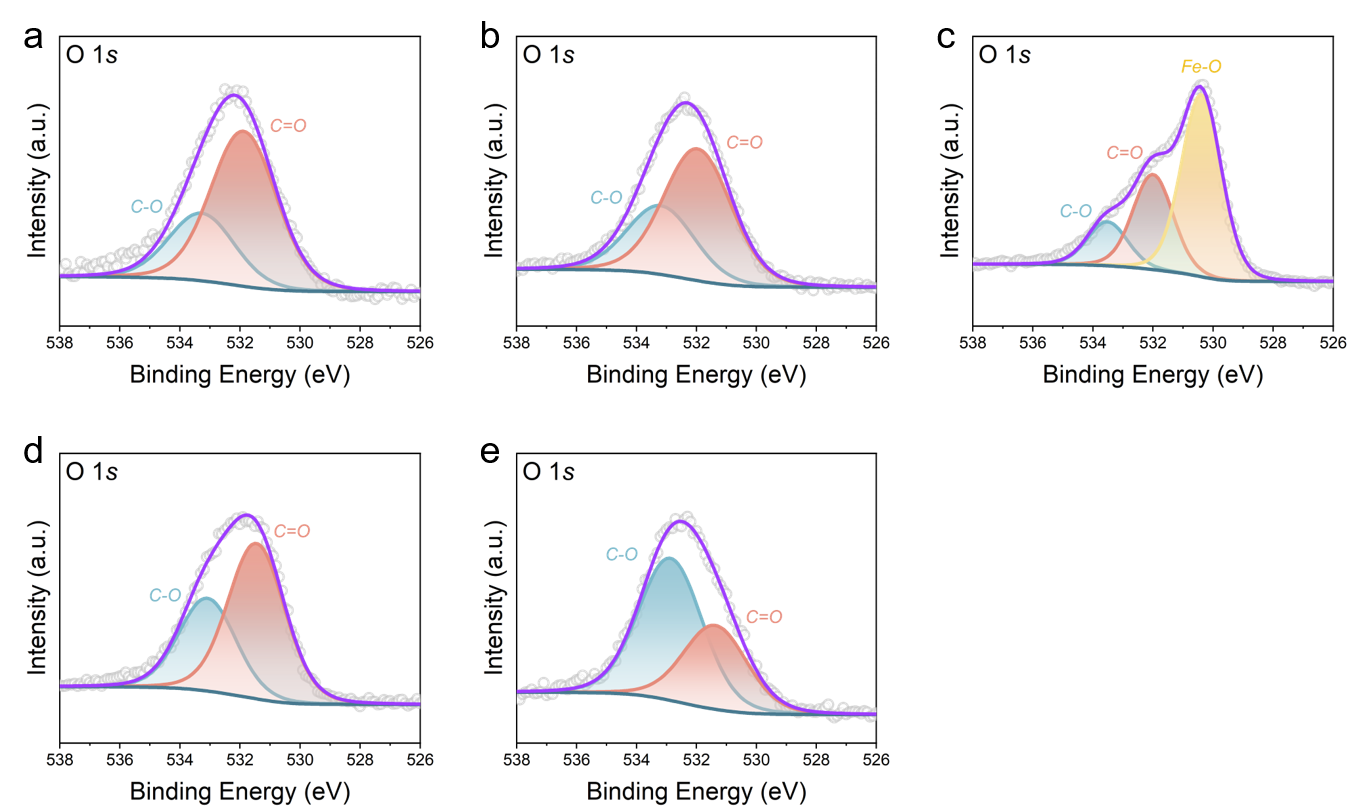


**Fig. S10** High-resolution XPS spectrum of O 1s spectra for a) NC, b) NC-Fe_AC1_, c) NC-Fe_AC2_, d) NC-Fe_AC3_, and e) NC-Fe_NP_


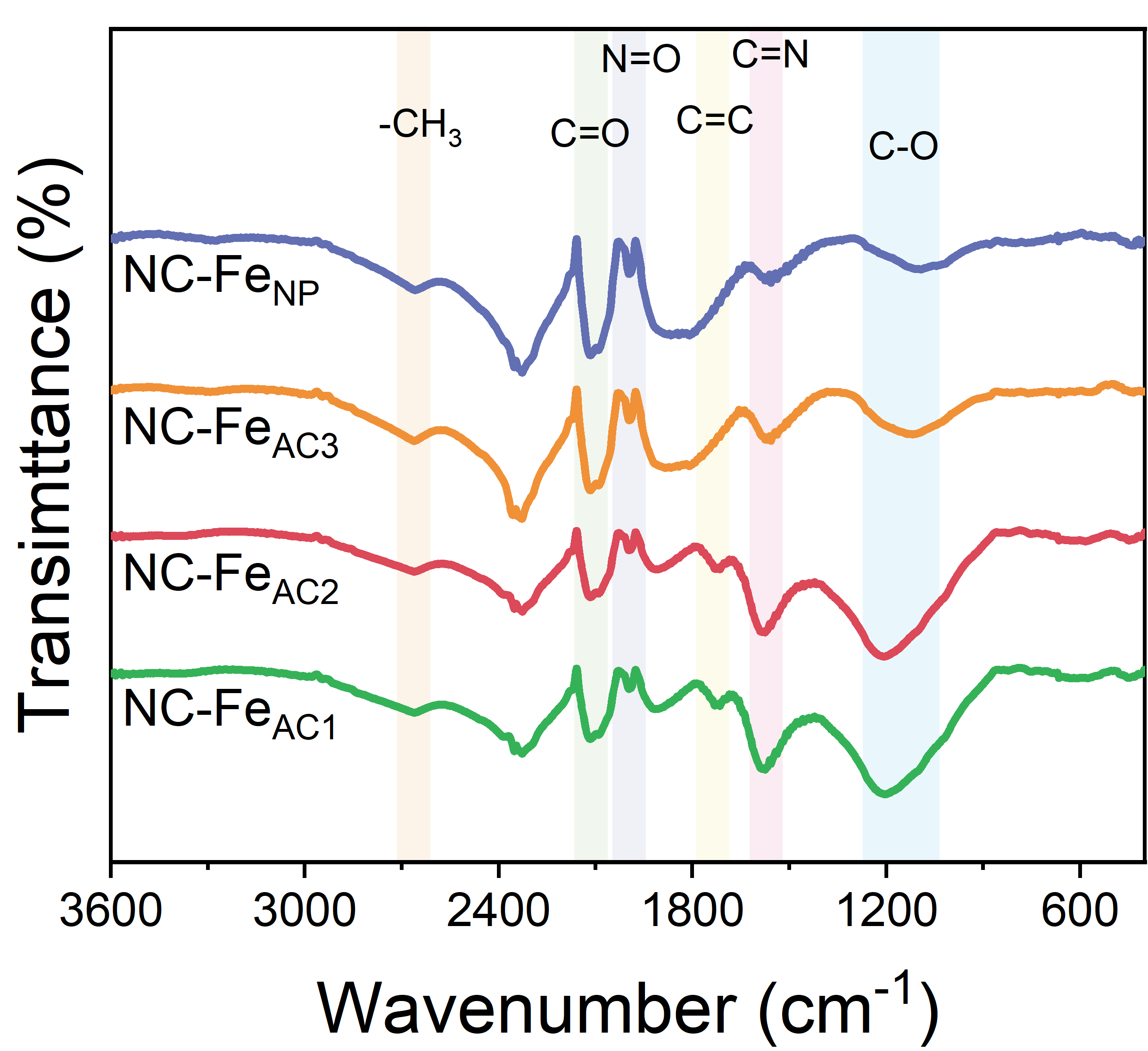


**Fig. S11** The FT-IR spectra of NC-Fe_ACX_, and NC-Fe_NP_


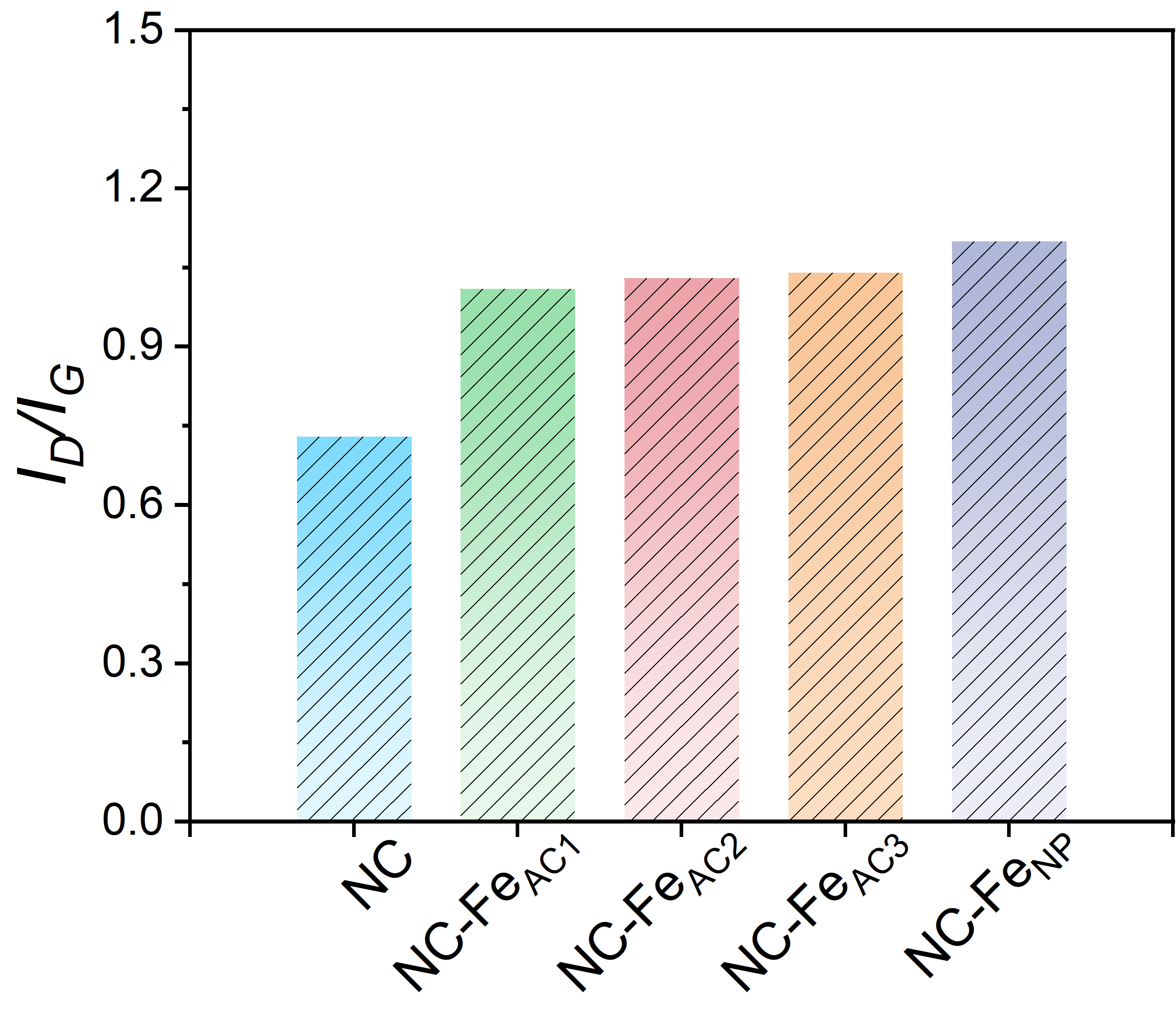


**Fig. S12** The *I_D_/I_G_* value of NC, NC-Fe_ACX_, and NC-Fe_NP_


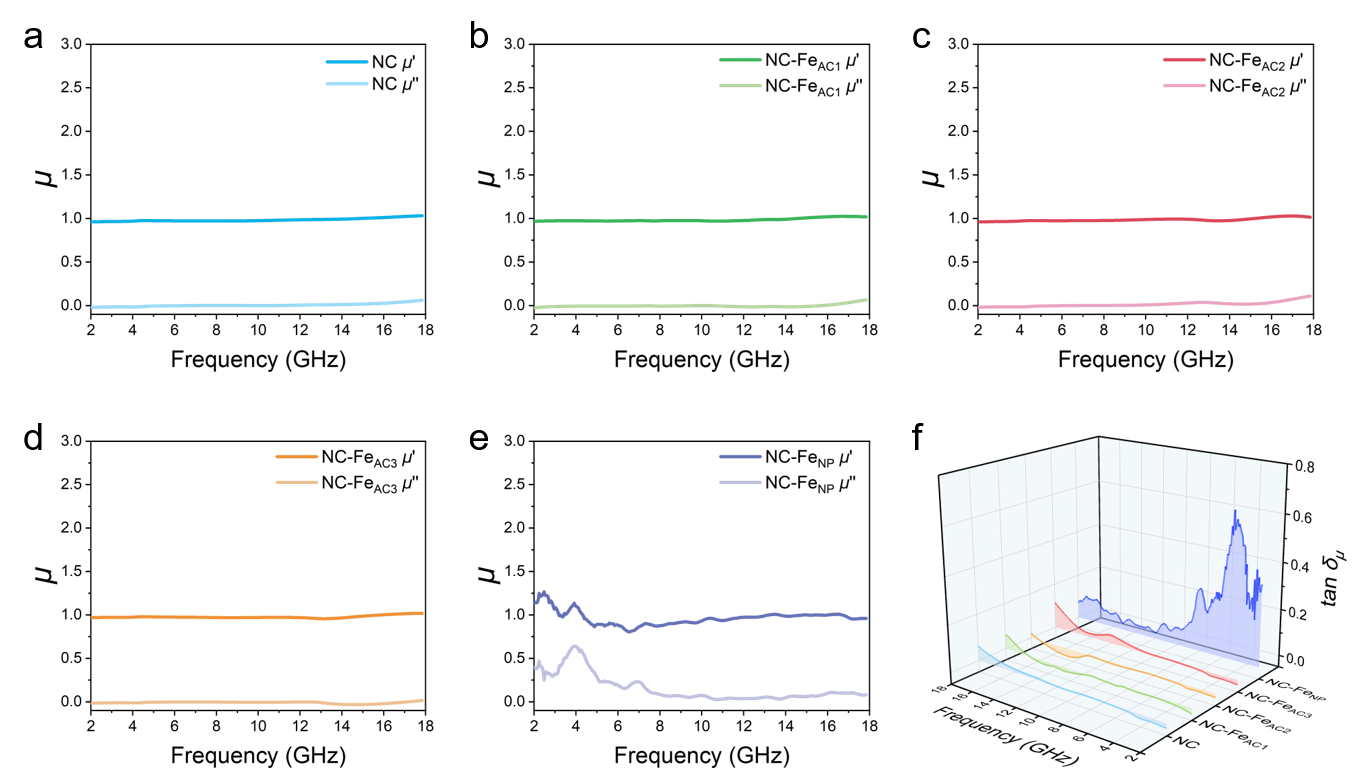


**Fig. S13** Real and imaginary parts of permeability for a) NC, b) NC-Fe_AC1_, c) NC-Fe_AC2_, d) NC-Fe_AC3_, and e) NC-Fe_NP_. f) Angular tangent of permeability curves of NC, NC-Fe_ACx_, and NC-Fe_NP_


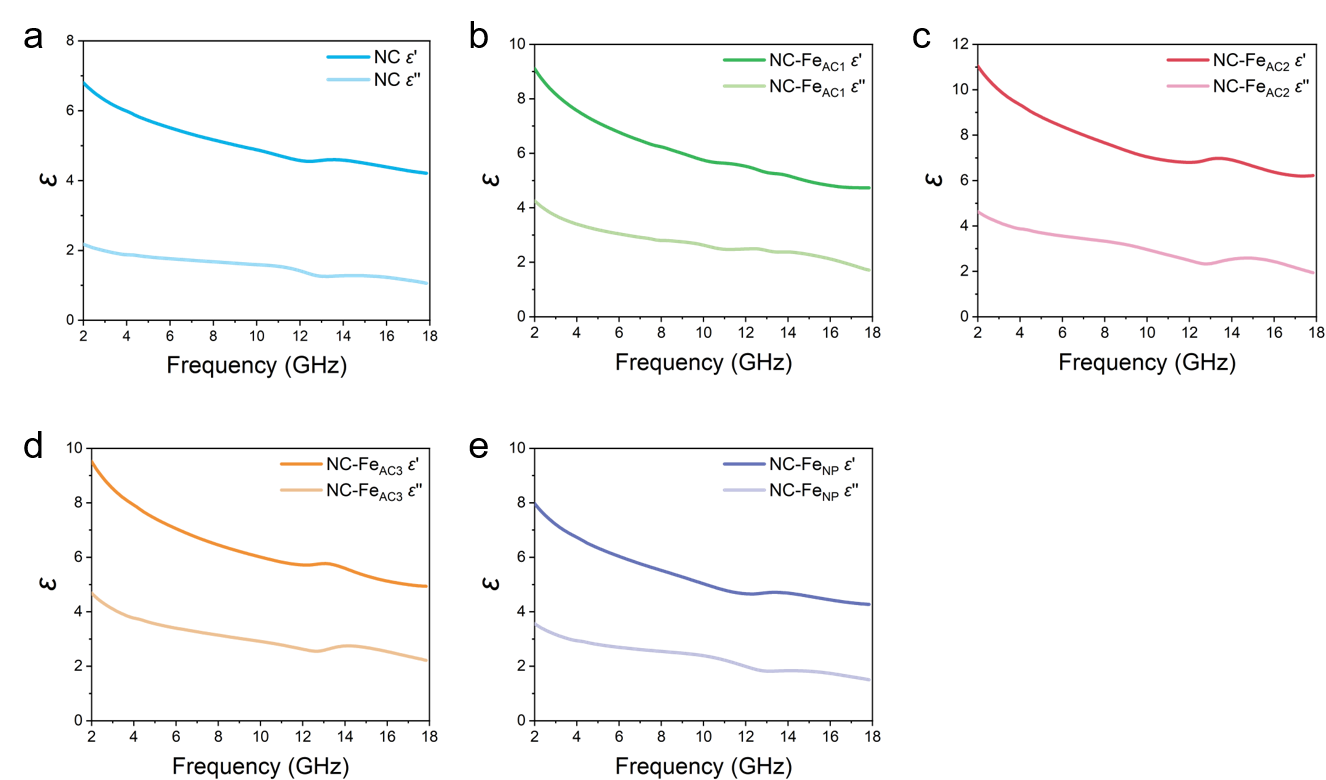


**Fig. S14** Real and imaginary parts of permittivity for a) NC, b) NC-Fe_AC1_, c) NC-Fe_AC2_, d) NC-Fe_AC3_, and e) NC-Fe_NP_


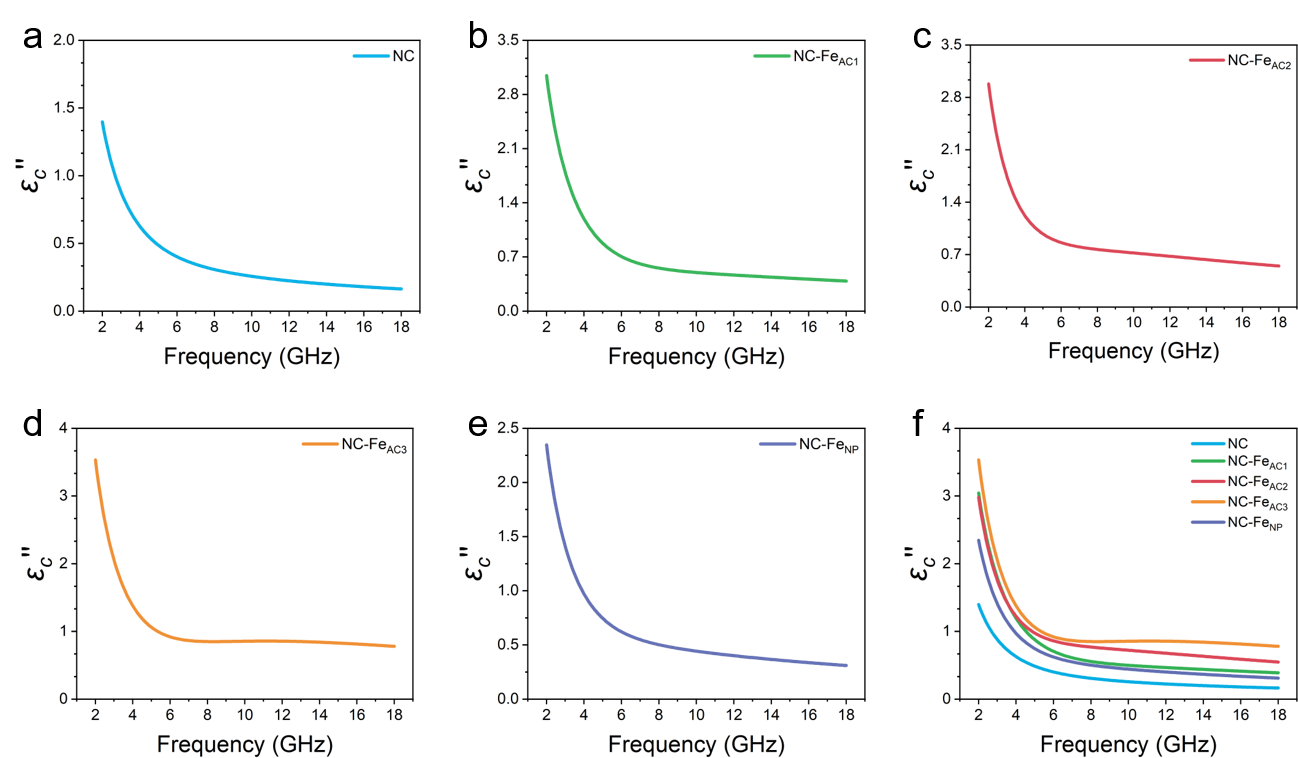


**Fig. S15** The contribution of conduction loss in a) NC, b) NC-Fe_AC1_, c) NC-Fe_AC2_, d) NC-Fe_AC3_, and e) NC-Fe_NP_. f) Comparison of conduction loss among samples


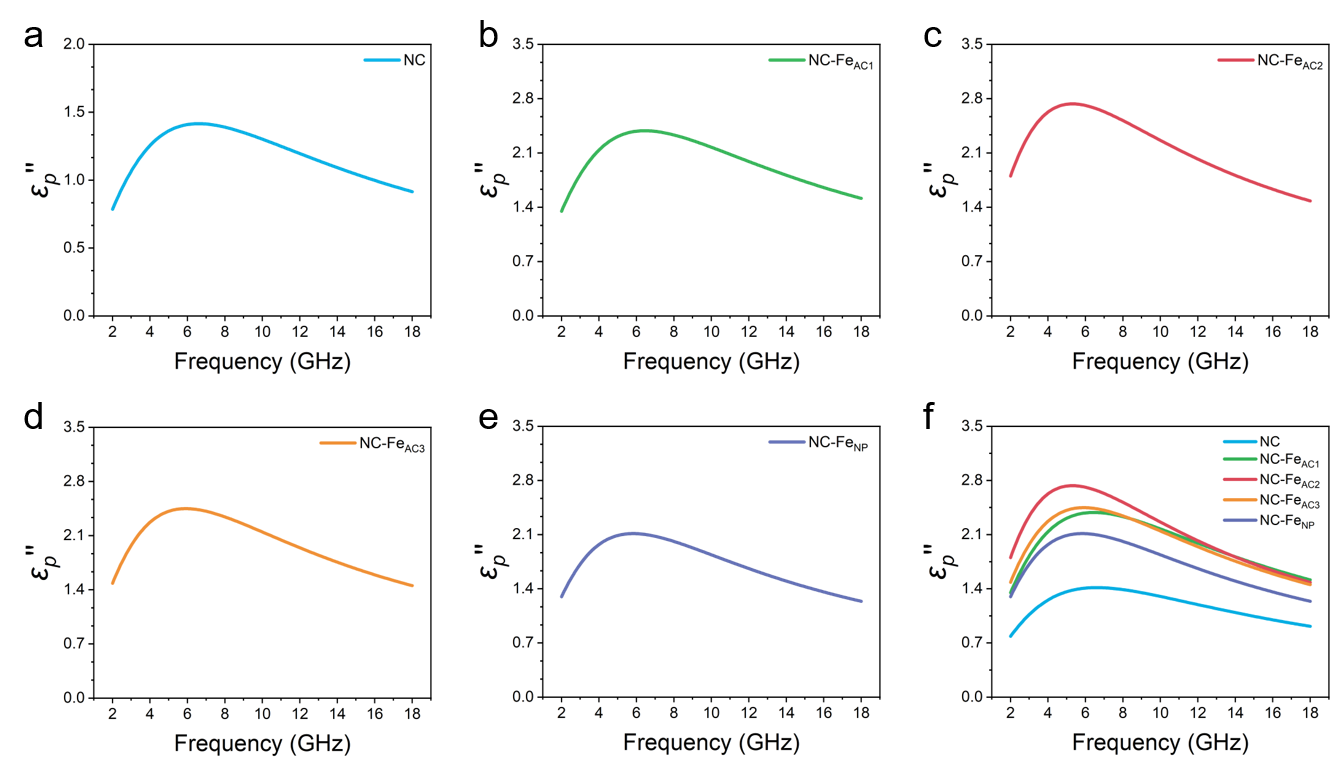


**Fig. S16** The contribution of polarization loss in a) NC, b) NC-Fe_AC1_, c) NC-Fe_AC2_, d) NC-Fe_AC3_, and e) NC-Fe_NP_. f) Comparison of polarization loss among samples


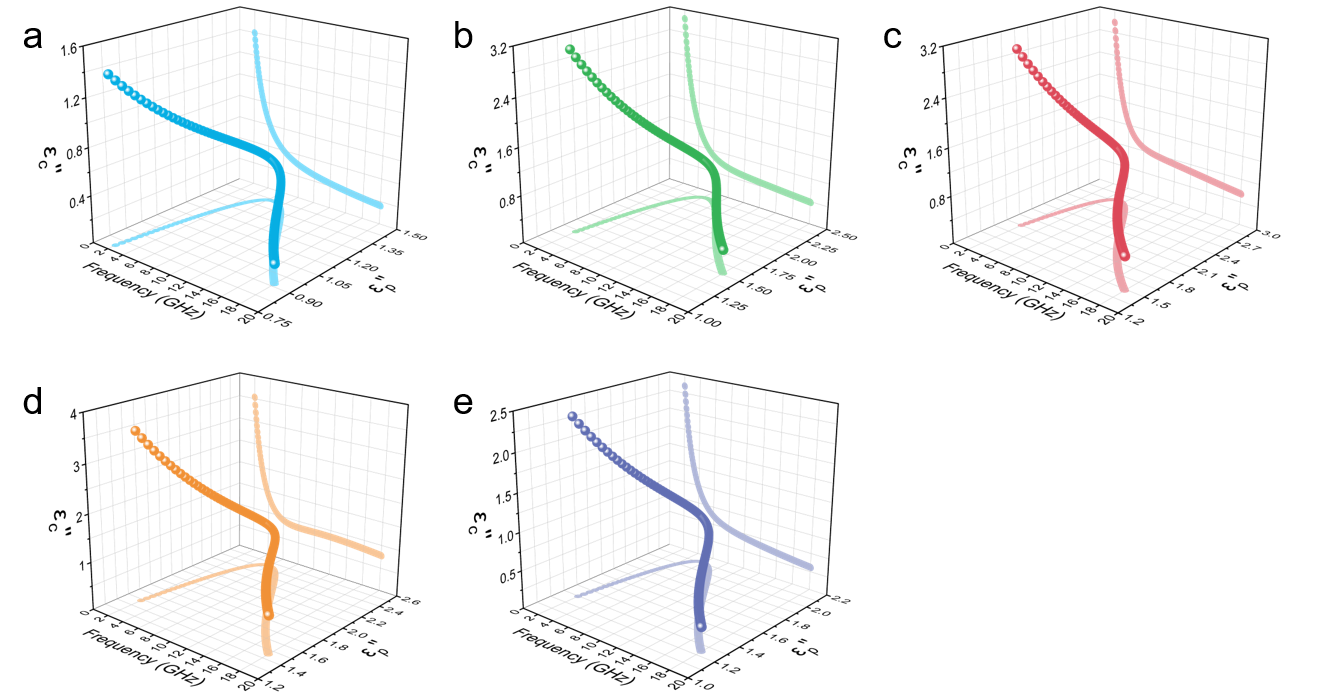


**Fig. S17** The relationship between the conduction and polarization loss with frequency of a) NC, b) NC-Fe_AC1_, c) NC-Fe_AC2_, d) NC-Fe_AC3_, and e) NC-Fe_NP_


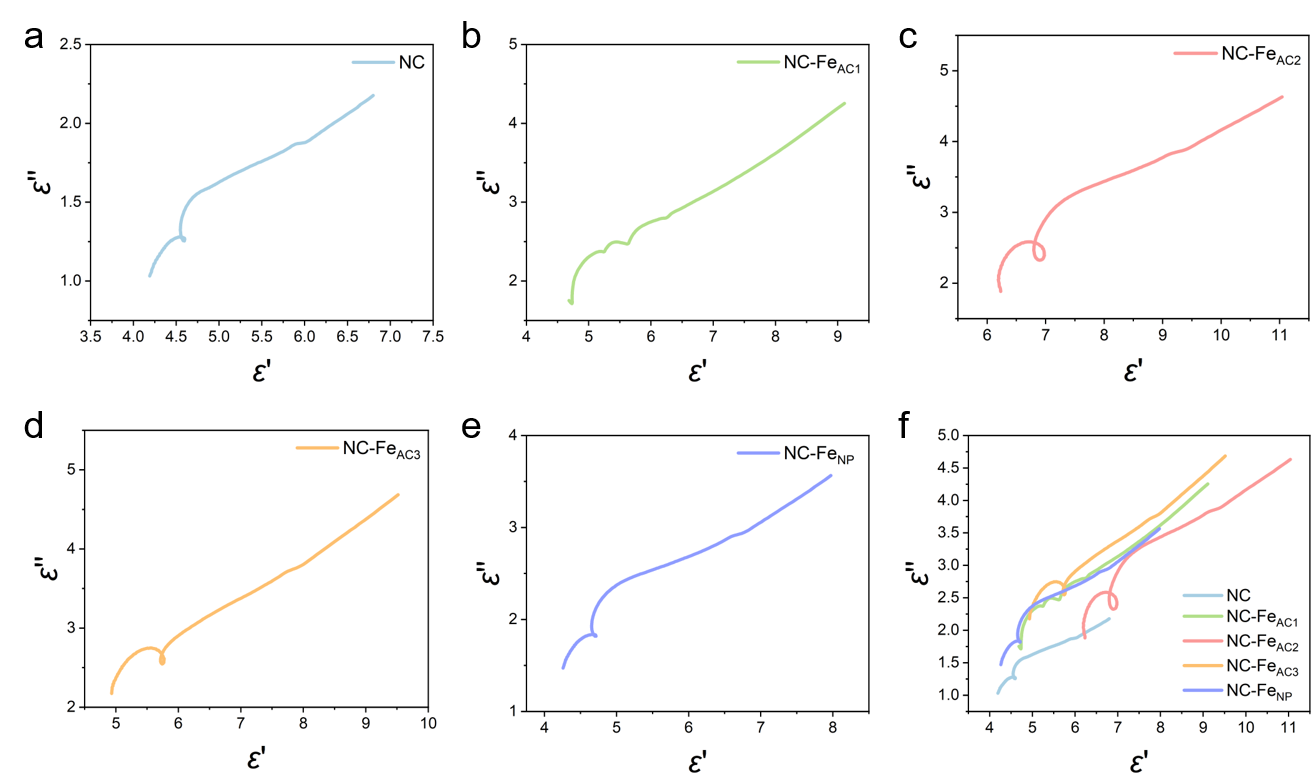


**Fig. S18** The Cole-Cole plot of a) NC, b) NC-Fe_AC1_, c) NC-Fe_AC2_, d) NC-Fe_AC3_, and e) NC-Fe_NP_. f) Comparison of Cole-Cole plot among samples


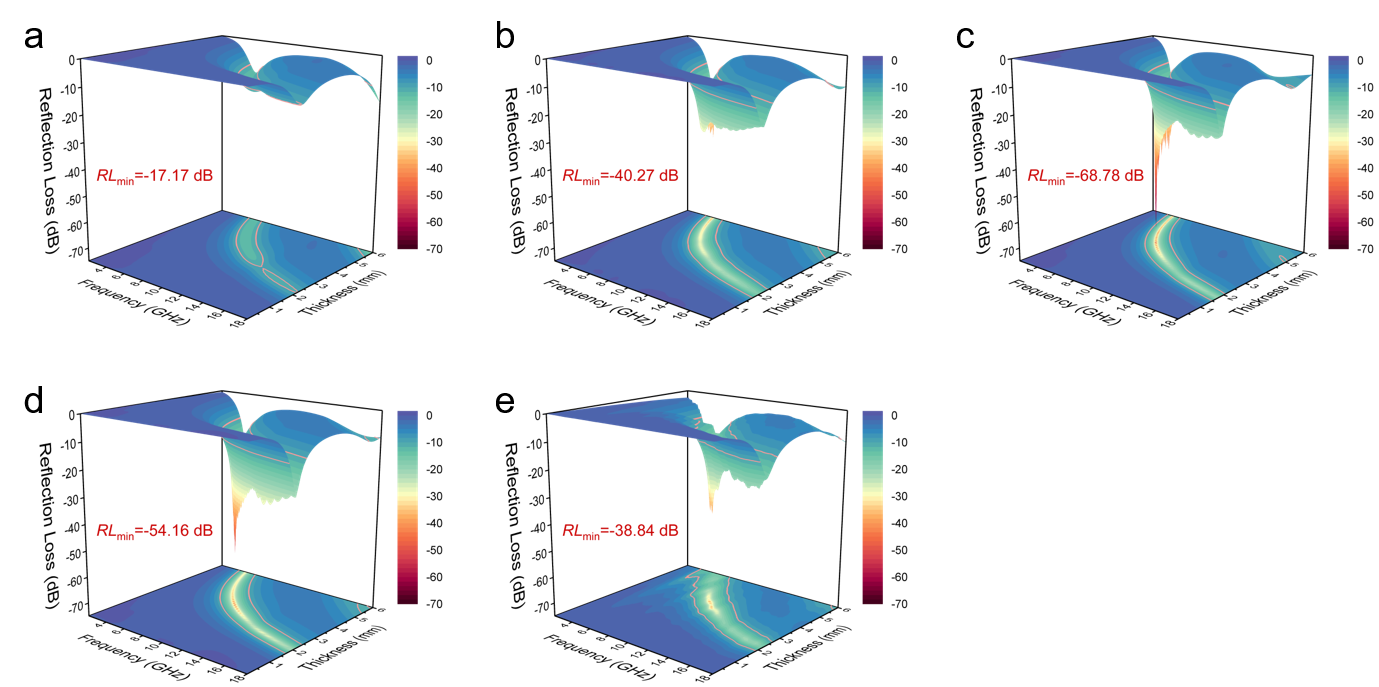


**Fig. S19** 3D reflection loss of a) NC, b) NC-Fe_AC1_, c) NC-Fe_AC2_, d) NC-Fe_AC3_, and e) NC-Fe_NP_


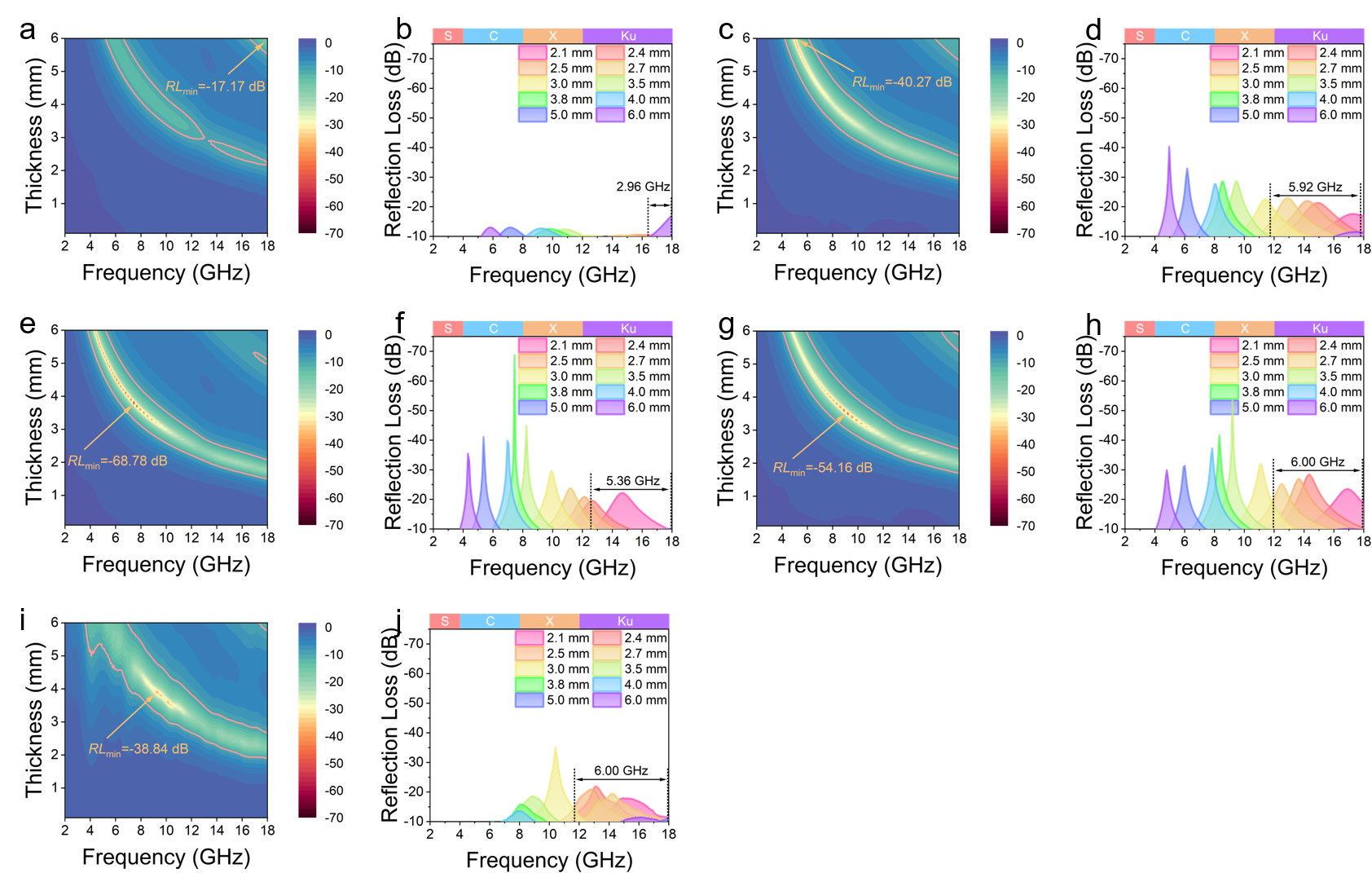


**Fig. S20** 2D reflection loss projection of a) NC, c) NC-Fe_AC1_, e) NC-Fe_AC2_, g) NC-Fe_AC3_, and i) NC-Fe_NP_. 2D reflection loss curves at different thickness of b) NC, d) NC-Fe_AC1_, f) NC-Fe_AC2_, h) NC-Fe_AC3_, and j) NC-Fe_NP_


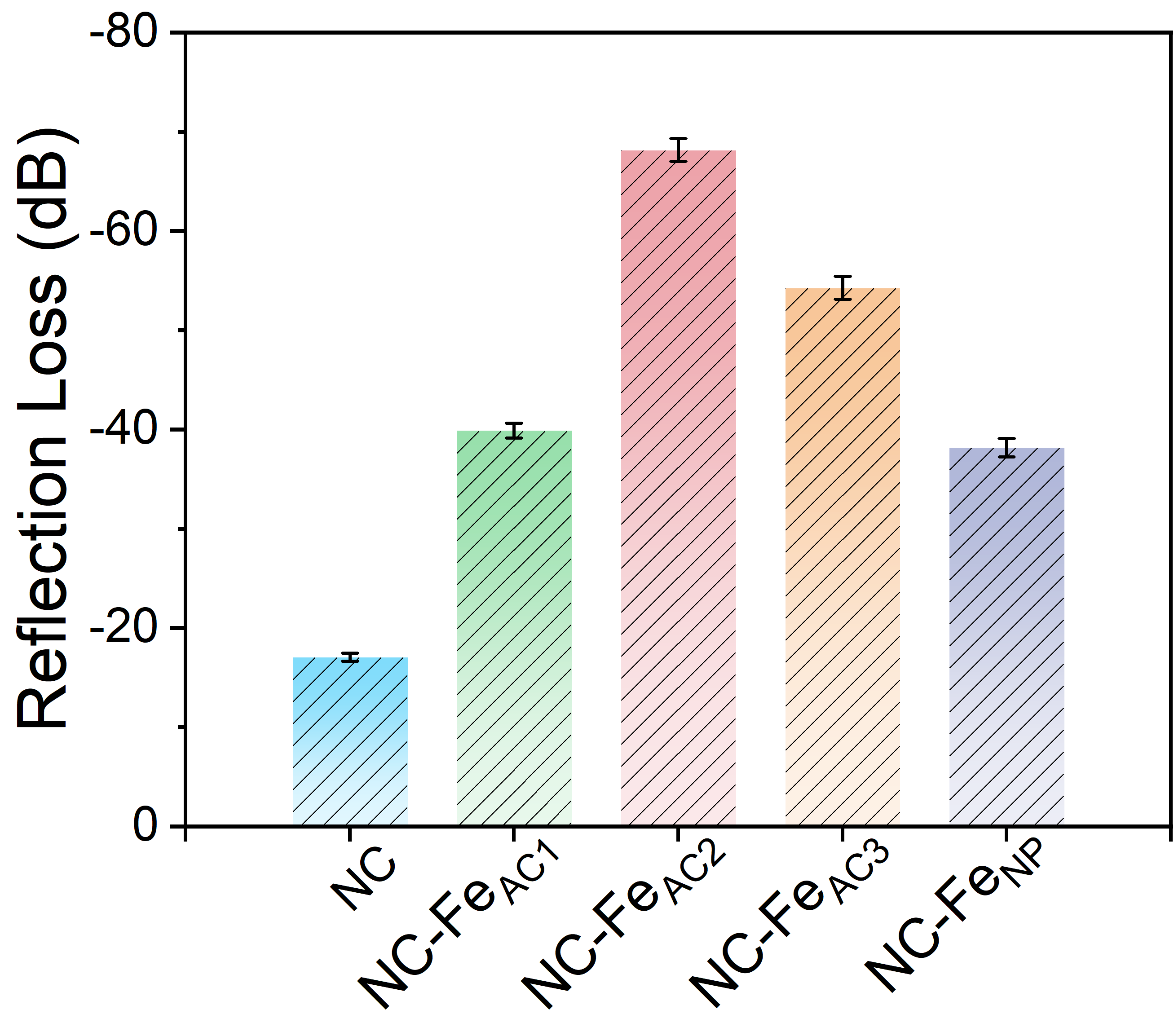


**Fig. S21** The electromagnetic wave absorption performance image of multiple measurements.

Average electromagnetic wave absorption performance obtained from five repeated measurements of NC, NC-Fe_ACX_, and NC-Fe_NP_ (6 wt.%)


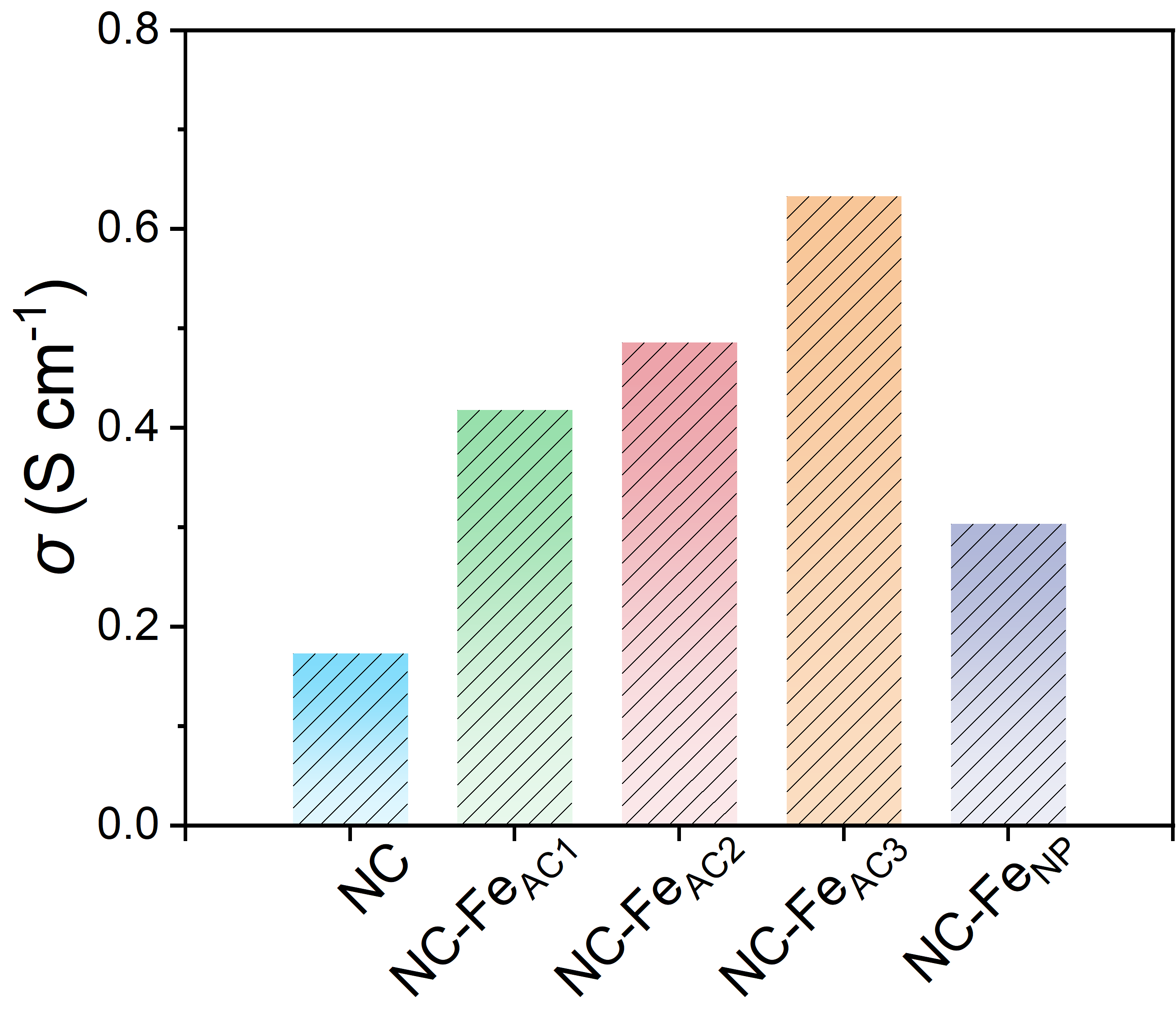


**Fig. S22** The *σ* value of NC, NC-Fe_ACX_, and NC-Fe_NP_ measured by four-probe method at 2 MPa

The electrical conductivity of the samples was measured using a ST2742B four-probe powder resistivity tester under a compressive pressure of 2 MPa. This experimentally observed trend agrees well with the conduction-related contribution inferred from our permittivity-based fitting of electromagnetic wave loss.


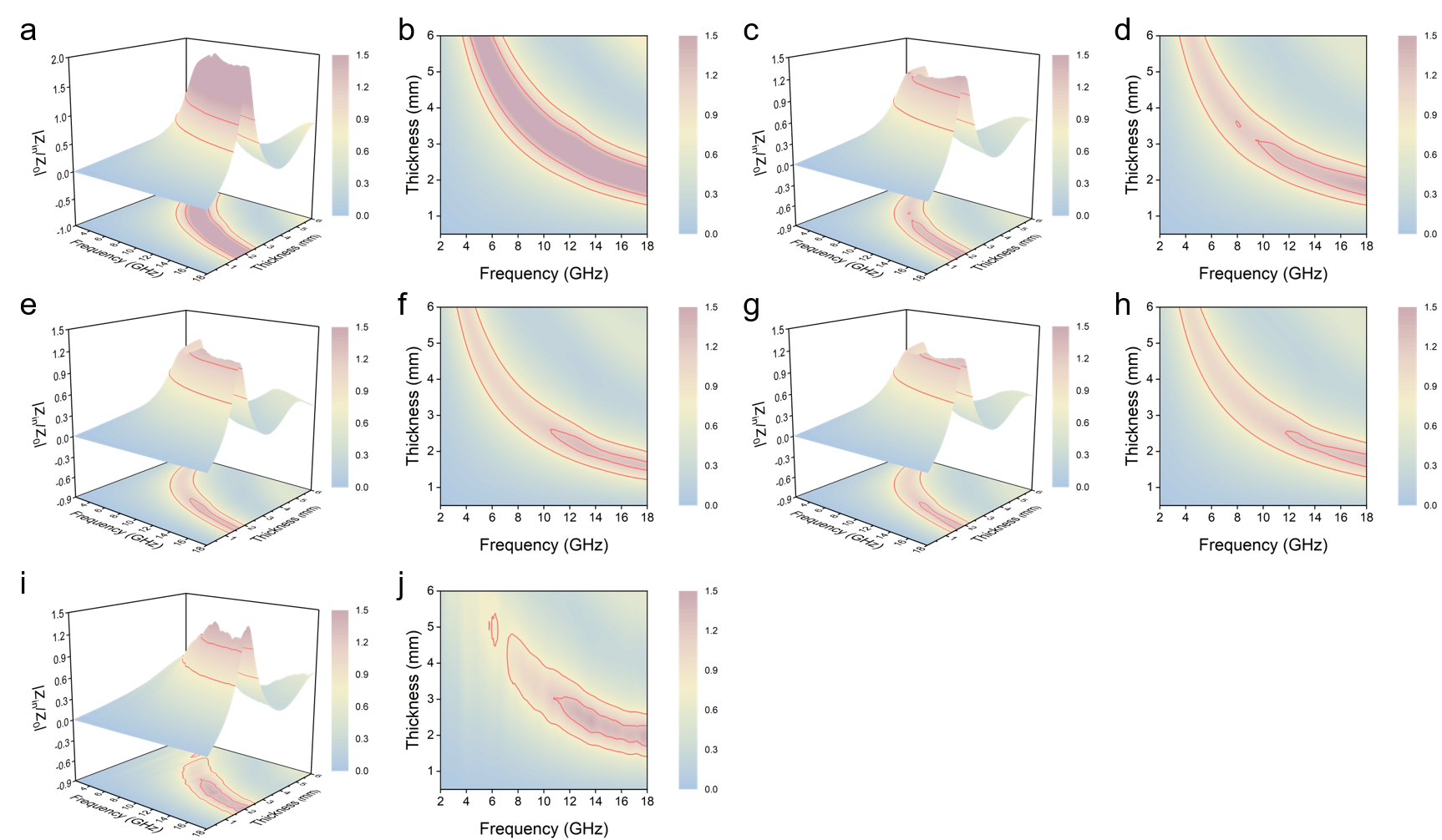


**Fig. S23** 3D impedance matching of a) NC, c) NC-Fe_AC1_, e) NC-Fe_AC2_, g) NC-Fe_AC3_, and i) NC-Fe_NP_. 2D impedance matching projection of b) NC, d) NC-Fe_AC1_, f) NC-Fe_AC2_, h) NC-Fe_AC3_, and j) NC-Fe_NP_


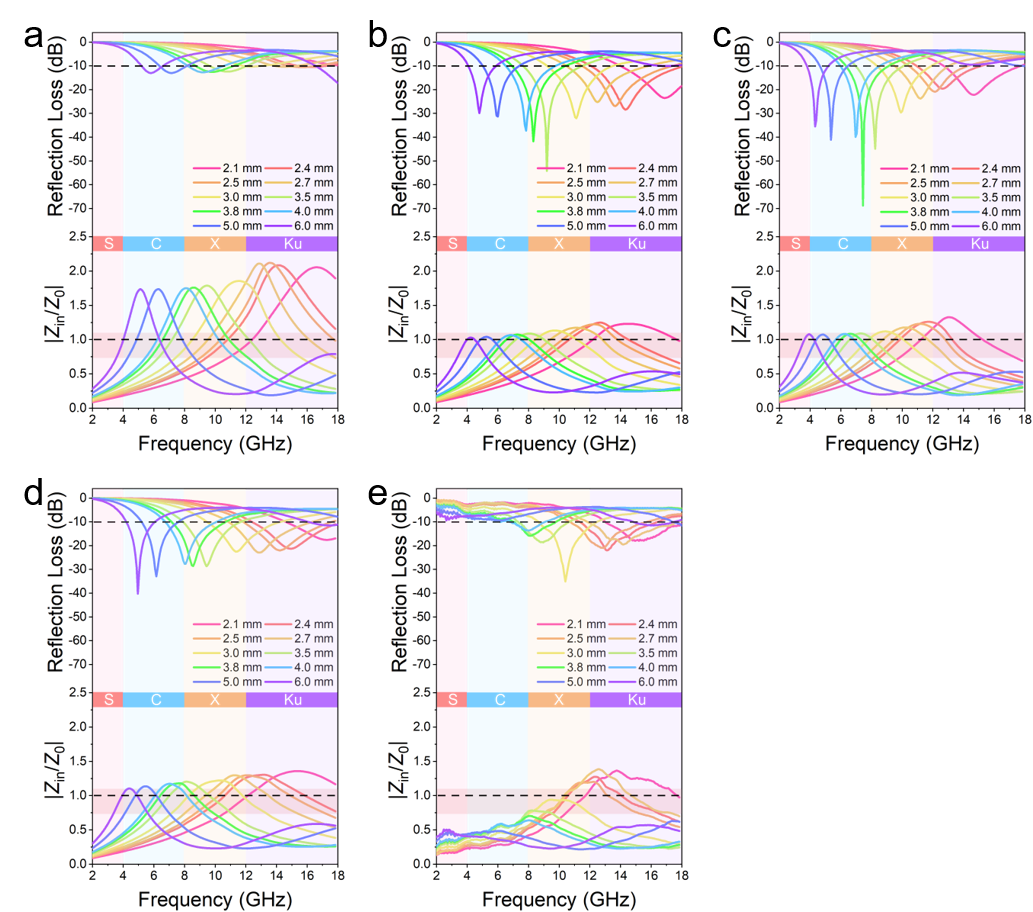


**Fig. S24** The Correspondence between 2D reflection loss and impedance matching curves at different thickness of a) NC, b) NC-Fe_AC1_, c) NC-Fe_AC2_, d) NC-Fe_AC3_, and e) NC-Fe_NP_


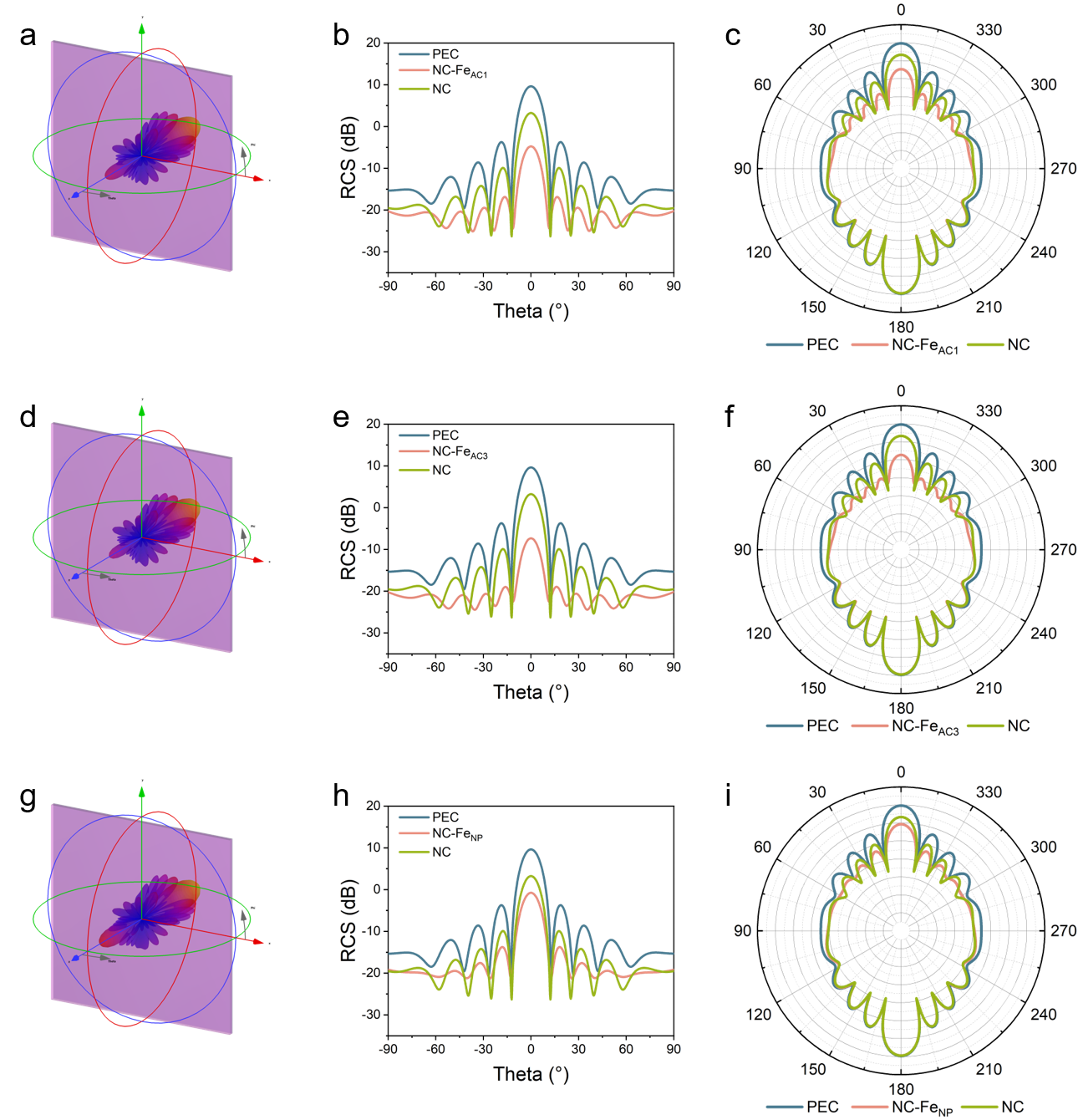


**Fig. S25** 3D radar wave scattering single of a) NC-Fe_AC1_, d) NC-Fe_AC3_, and g) NC-Fe_NP_. RCS simulation curves of b, c) NC-Fe_AC1_, e, f) NC-Fe_AC3_, and h, i) NC-Fe_NP_


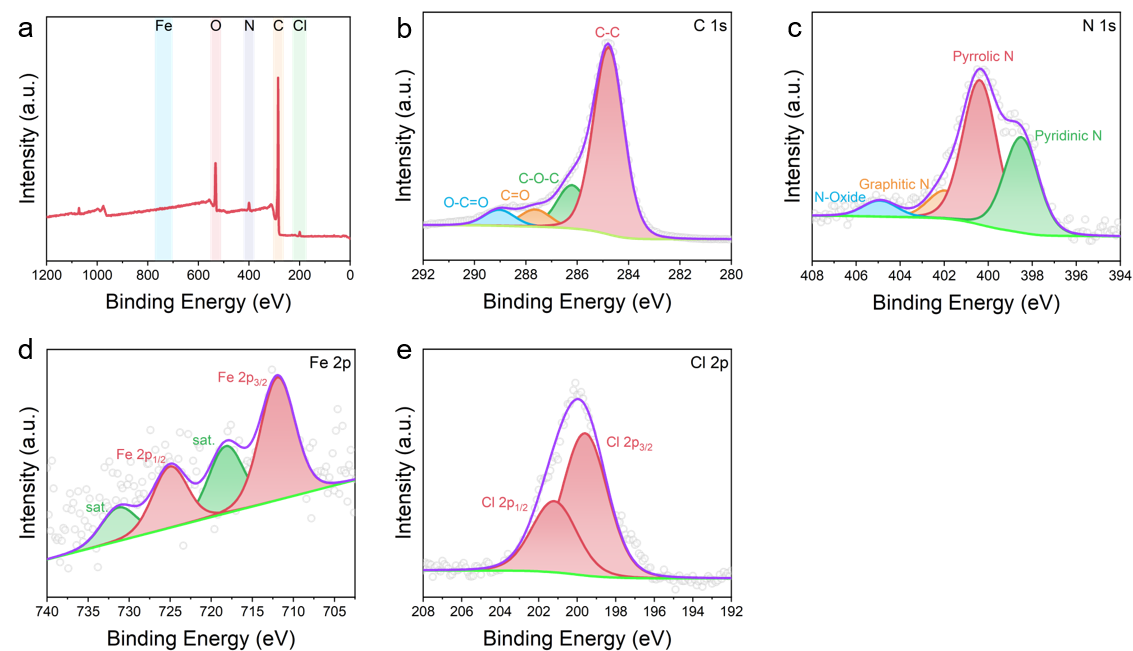


**Fig. S26** a) XPS survey spectra. b) C 1s, c) N 1s, d) Fe 2p, and e) Cl 2p high-resolution XPS spectra of NC-Fe_AC2_ after one-month corrosion

The XPS survey spectrum clearly reveals the presence of Cl element (Fig. S26a), the high-resolution Cl *2p* spectrum (Fig.S26e) shows a Cl 2p_3/2_ component at ~199.5 eV, which can be assigned to metal-chloride (M-Cl) species, indicating that Fe clusters preferentially adsorb/bind Cl^‒^. Meanwhile, the C *1s* spectrum (Fig. S26b) can be deconvoluted into contributions from C-C, C-O-C, C=O, and O-C=O bonds, and the N *1s* spectrum (Fig.S26c) consists of pyridinic N, pyrrolic N, graphitic N, and N-O species; both spectra exhibit no pronounced changes compared with those before corrosion, suggesting that the carbon framework and N-coordination environment remain largely intact. Notably, the Fe *2p* peaks (Fig.S26d) show a slight shift toward higher binding energies, implying an increased average oxidation state of Fe, which is consistent with electron withdrawal induced by chloride coordination predominantly on Fe cluster domains.


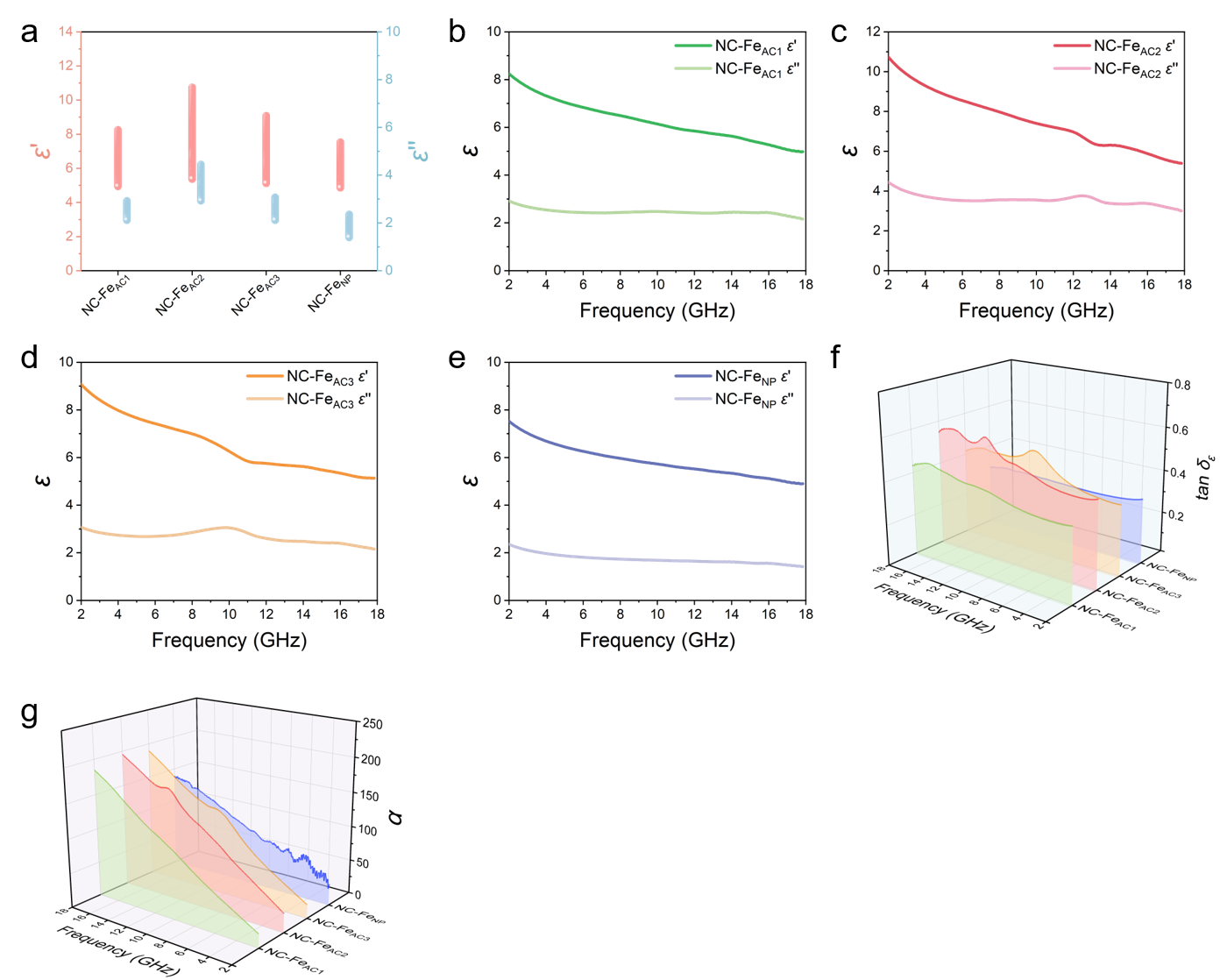


**Fig. S27** a) The range of real and imaginary permittivity of samples after NaCl treatment. Real and imaginary parts of permittivity for b) NC-Fe_AC1_, c) NC-Fe_AC2_, d) NC-Fe_AC3_, and e) NC-Fe_NP_ after NaCl treatment. f) Angular tangent of permittivity curves of NC, NC-Fe_ACx_, and NC-Fe_NP_ after NaCl treatment. g) The attenuation constant of samples after NaCl treatment


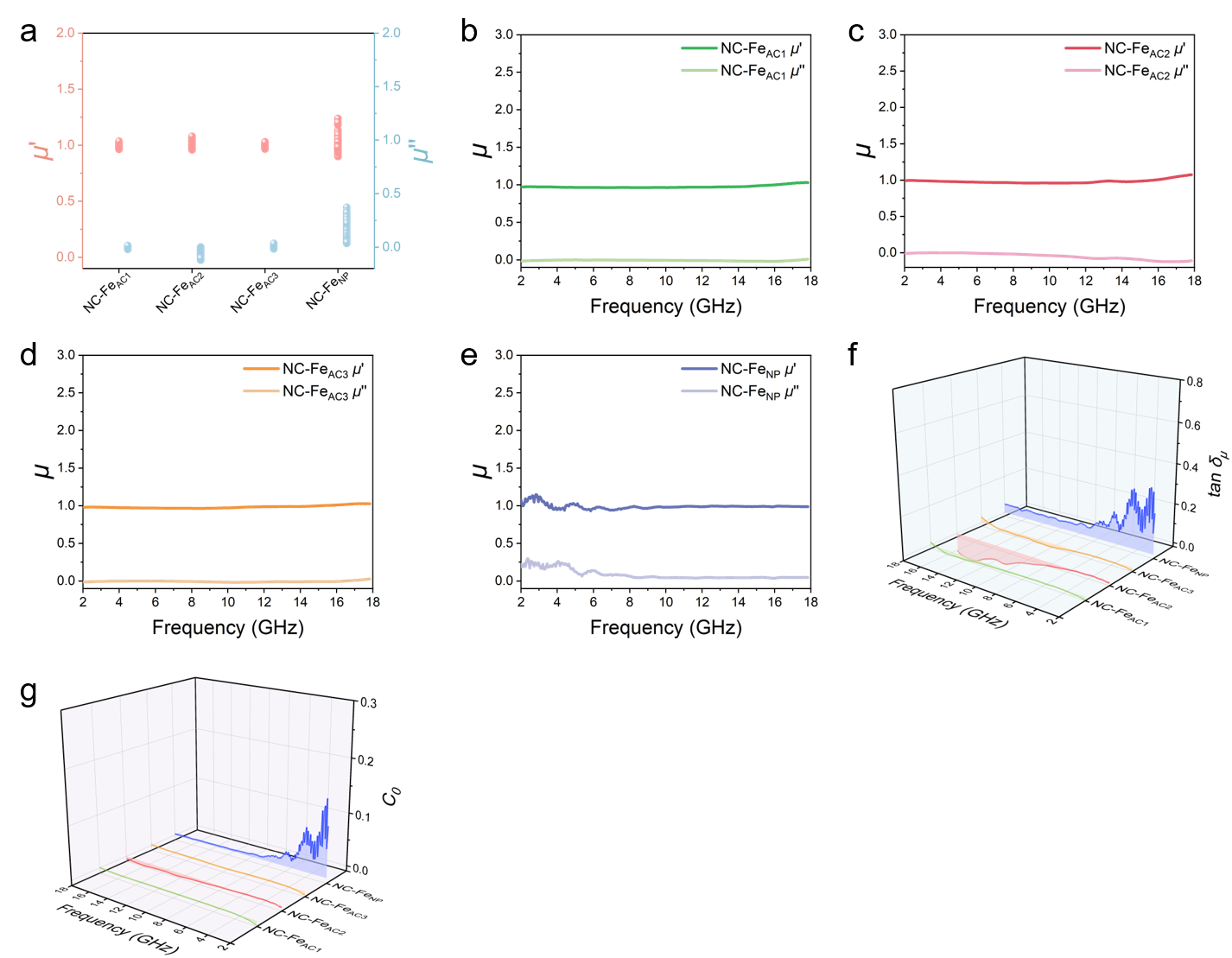


**Fig. S28** a) The range of real and imaginary permeability of samples after NaCl treatment. Real and imaginary parts of permeability for b) NC-Fe_AC1_, c) NC-Fe_AC2_, d) NC-Fe_AC3_, and e) NC-Fe_NP_ after NaCl treatment. f) Angular tangent of permeability curves of NC, NC-Fe_ACx_, and NC-Fe_NP_ after NaCl treatment. g) The *C_0_* value of samples after NaCl treatment


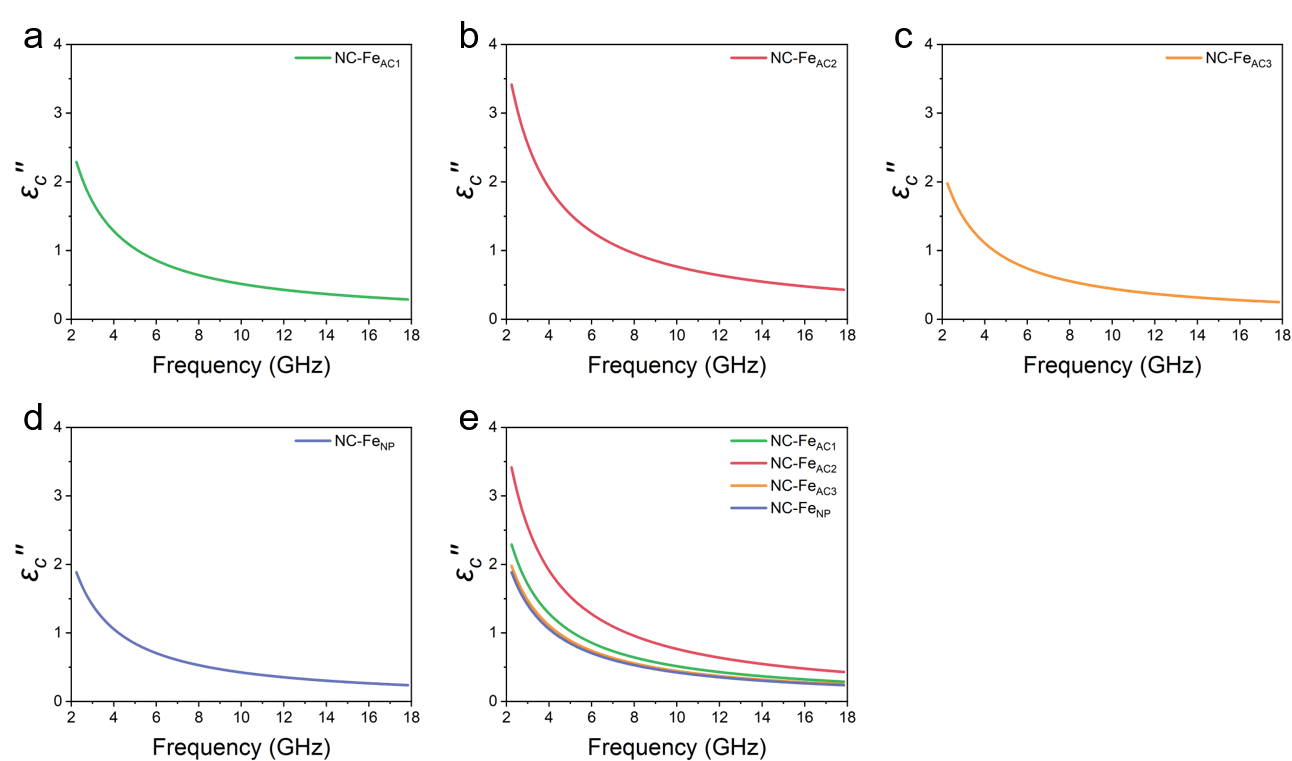


**Fig. S29** The contribution of conduction loss in a) NC, b) NC-Fe_AC1_, c) NC-Fe_AC2_, d) NC-Fe_AC3_, and e) NC-Fe_NP_ after NaCl treatment. f) Comparison of conduction loss among samples after NaCl treatment


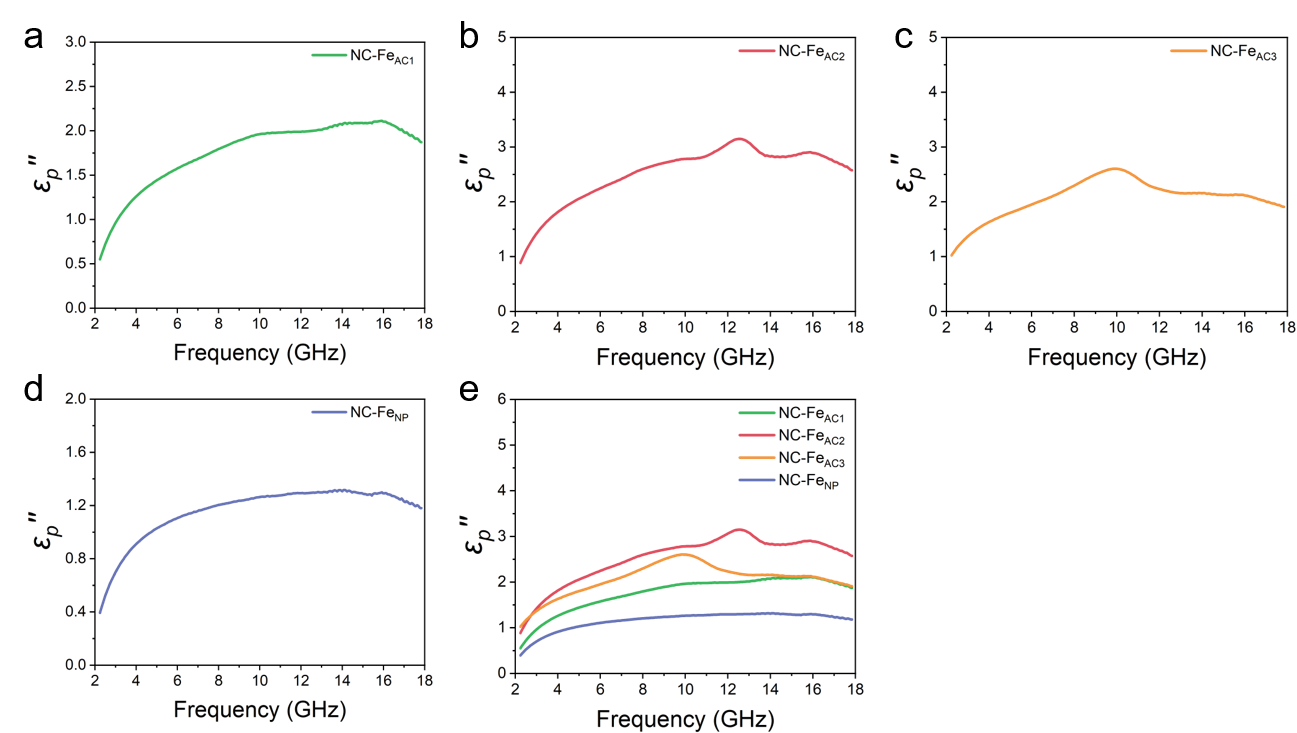


**Fig. S30** The contribution of polarization loss in a) NC, b) NC-Fe_AC1_, c) NC-Fe_AC2_, d) NC-Fe_AC3_, and e) NC-Fe_NP_ after NaCl treatment. f) Comparison of polarization loss among samples after NaCl treatment


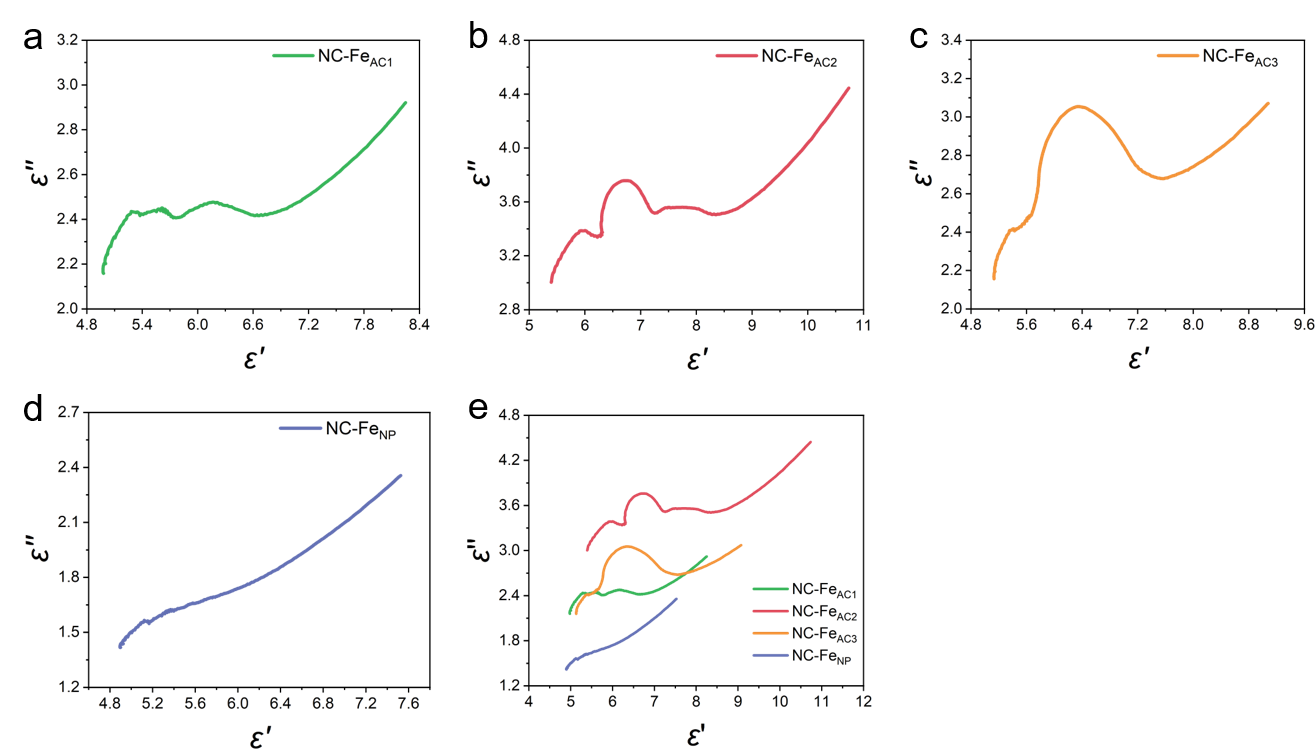


**Fig. S31** The Cole-Cole plot of a) NC-Fe_AC1_, b) NC-Fe_AC2_, c) NC-Fe_AC3_, and d) NC-Fe_NP_ after NaCl treatment. e) Comparison of Cole-Cole plot among samples after NaCl treatment


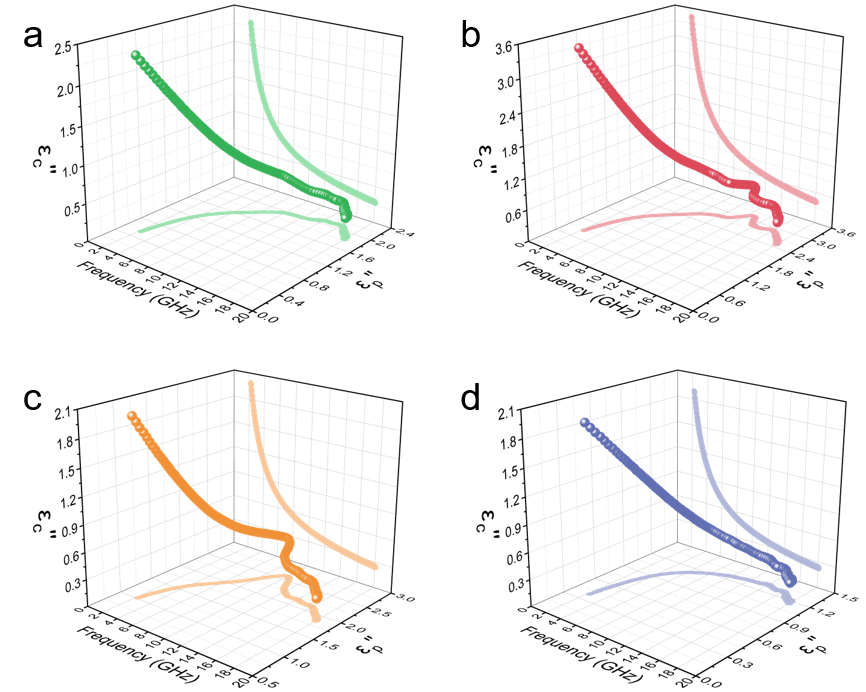


**Fig. S32** The relationship between the conduction and polarization loss with frequency of a) NC-Fe_AC1_, b) NC-Fe_AC2_, c) NC-Fe_AC3_, and d) NC-Fe_NP_ after NaCl treatment


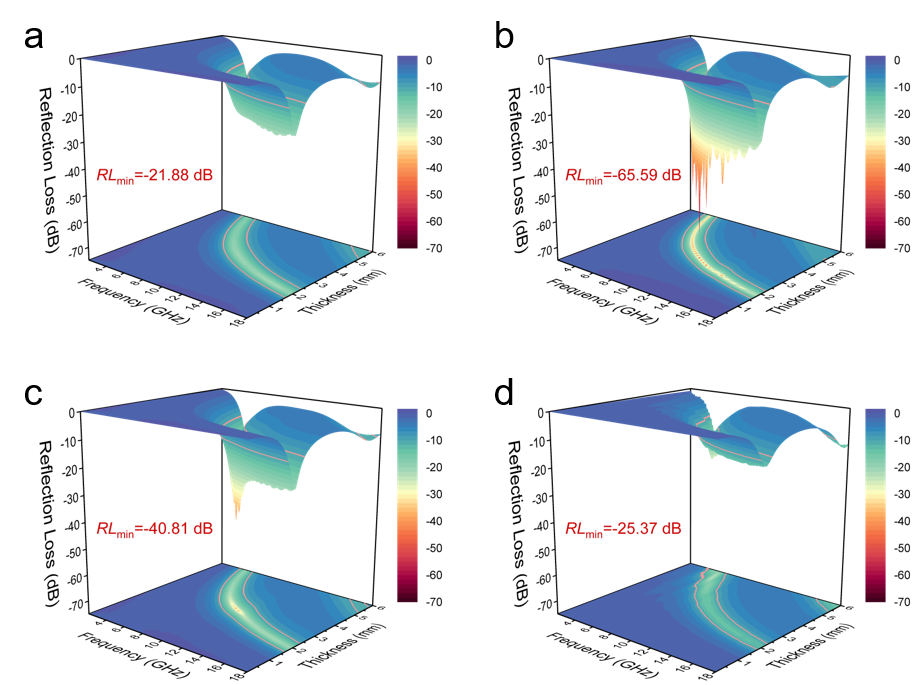


**Fig. S33** 3D reflection loss of a) NC-Fe_AC1_, b) NC-Fe_AC2_, c) NC-Fe_AC3_, and d) NC-Fe_NP_ after NaCl treatment


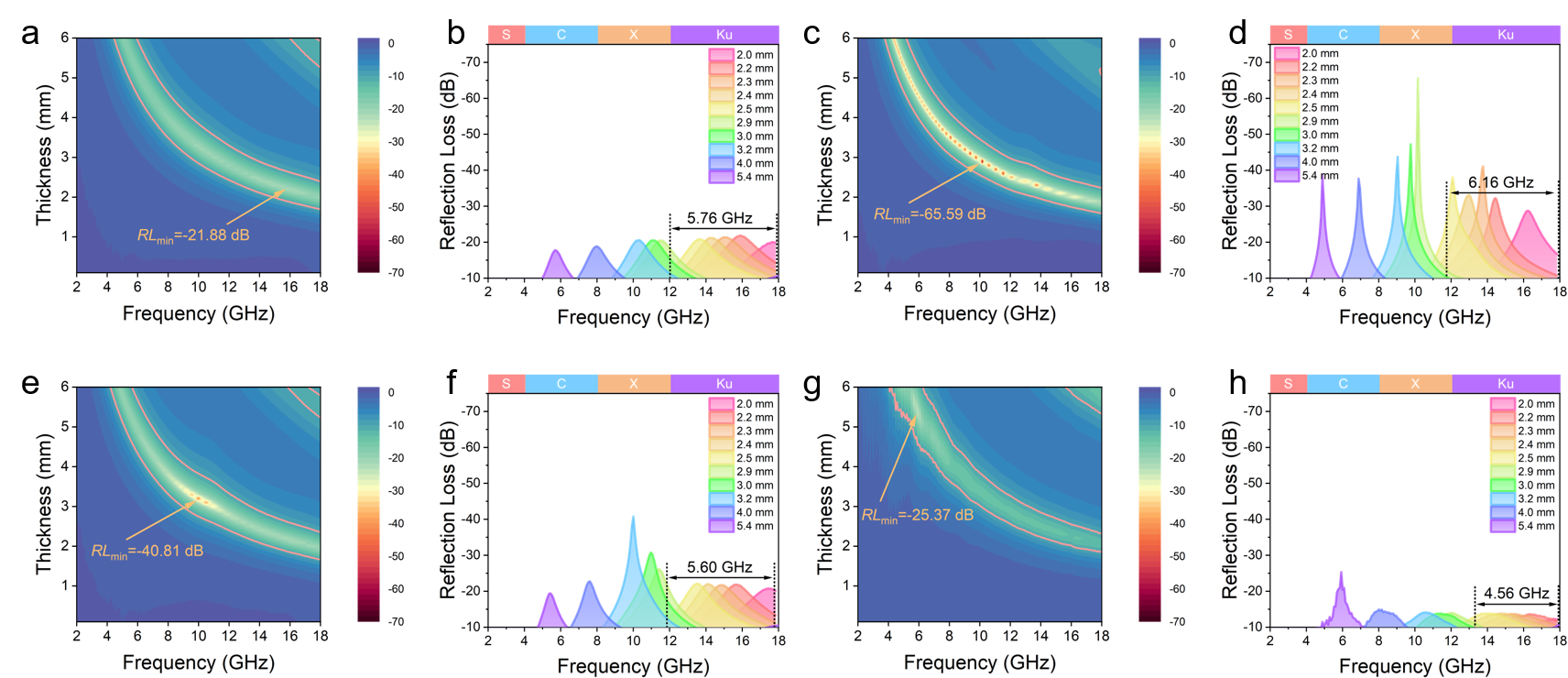


**Fig. S34** 2D reflection loss projection of a) NC-Fe_AC1_, c) NC-Fe_AC2_, e) NC-Fe_AC3_, and g) NC-Fe_NP_ after NaCl treatment. 2D reflection loss curves at different thickness of b) NC-Fe_AC1_, d) NC-Fe_AC2_, f) NC-Fe_AC3_, and h) NC-Fe_NP_ after NaCl treatment


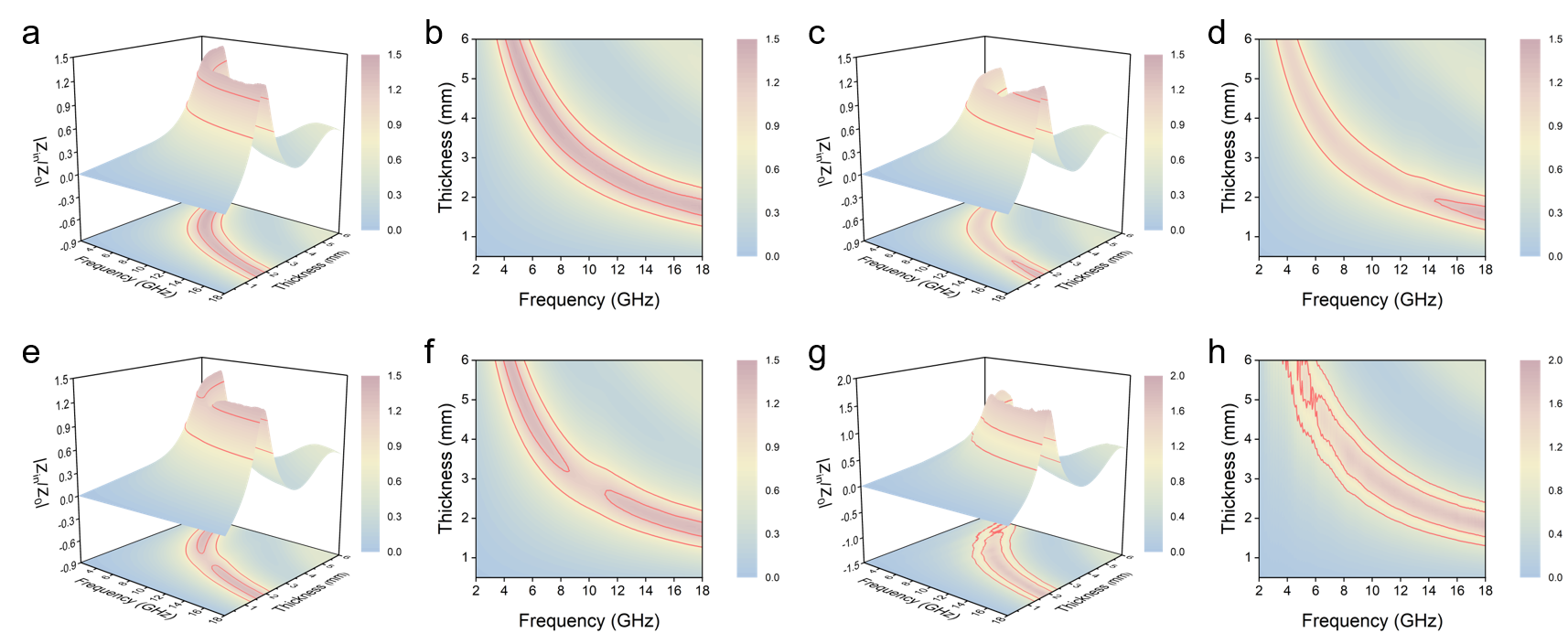


**Fig. S35** 3D impedance matching of a) NC-Fe_AC1_, c) NC-Fe_AC2_, e) NC-Fe_AC3_, and g) NC-Fe_NP_ after NaCl treatment. 2D impedance matching projection of b) NC-Fe_AC1_, d) NC-Fe_AC2_, f) NC-Fe_AC3_, and h) NC-Fe_NP_ after NaCl treatment


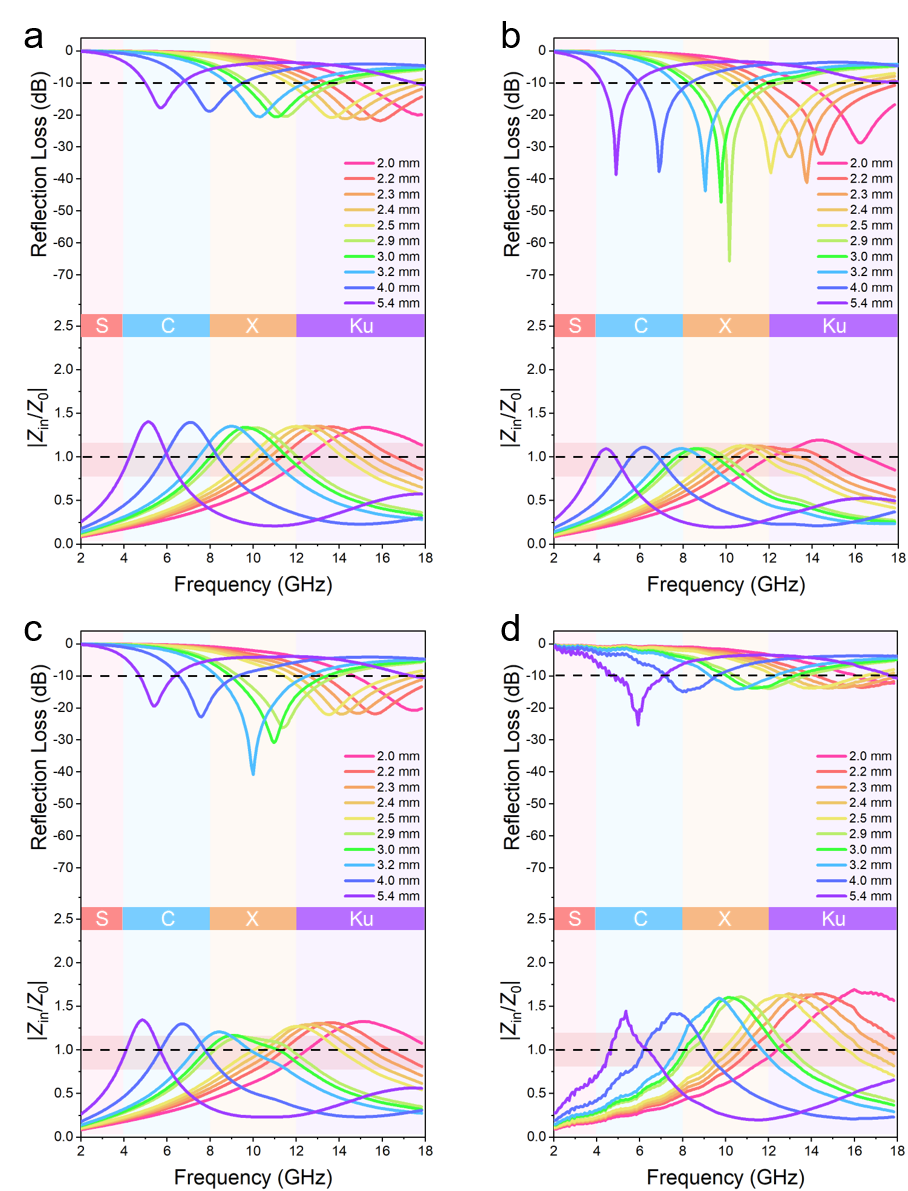


**Fig. S36** The Correspondence between 2D reflection loss and impedance matching curves at different thickness of a) NC, b) NC-Fe_AC1_, c) NC-Fe_AC2_, d) NC-Fe_AC3_, and e) NC-Fe_NP_ after NaCl treatment


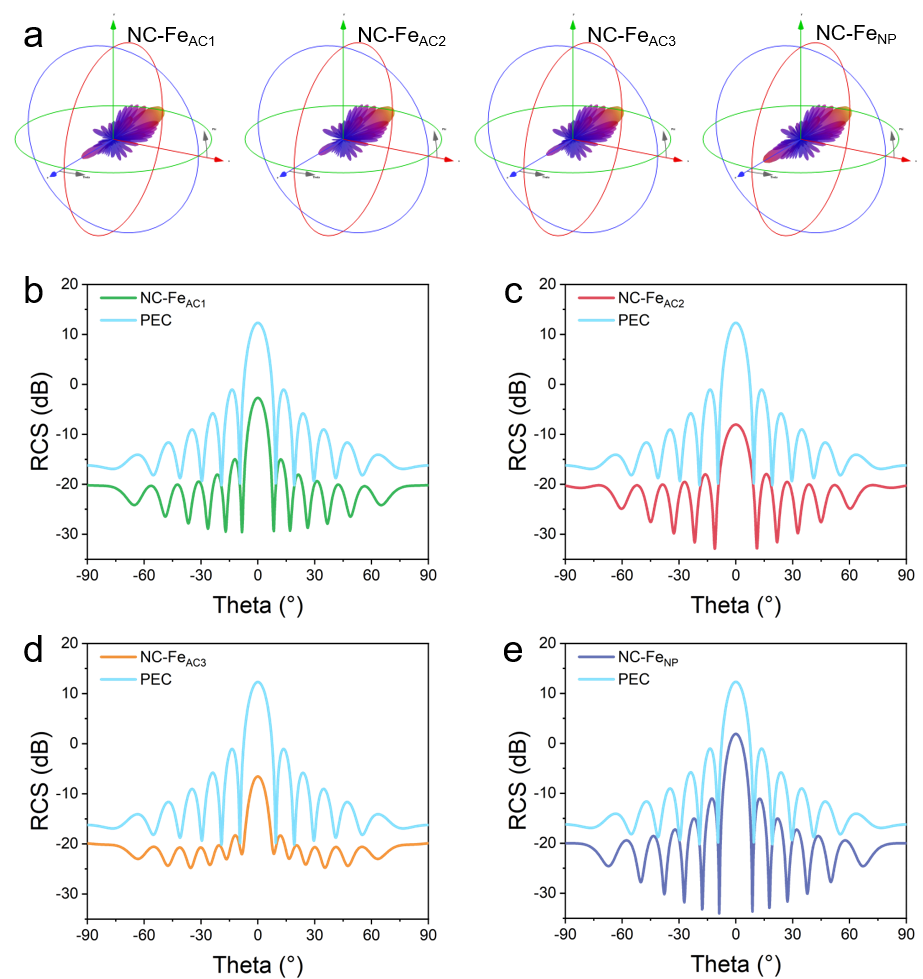


**Fig. S37** a) 3D RCS simulation curves of NC-Fe_ACX_, and NC-Fe_NP_. 2D RCS simulation curves of b) NC-Fe_AC1_, c) NC-Fe_AC2_, d) NC-Fe_AC3_ and e) NC-Fe_NP_


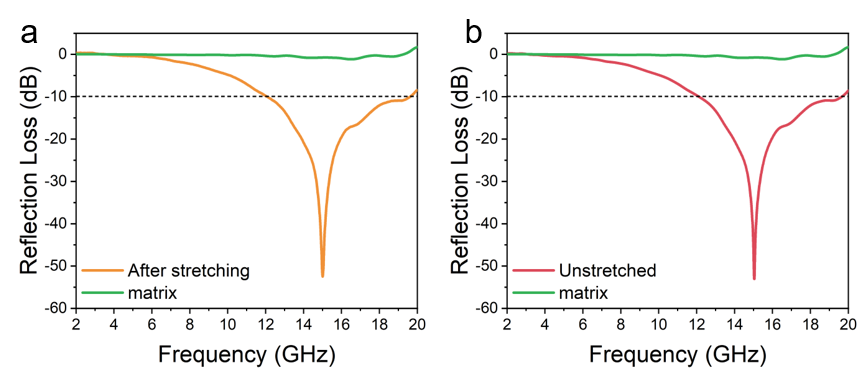


**Fig. S38** EMW performance of a) matrix and stretched NC-Fe_AC2_ film and b) matrix and unstretched NC-Fe_AC2_ film measured by bow-method


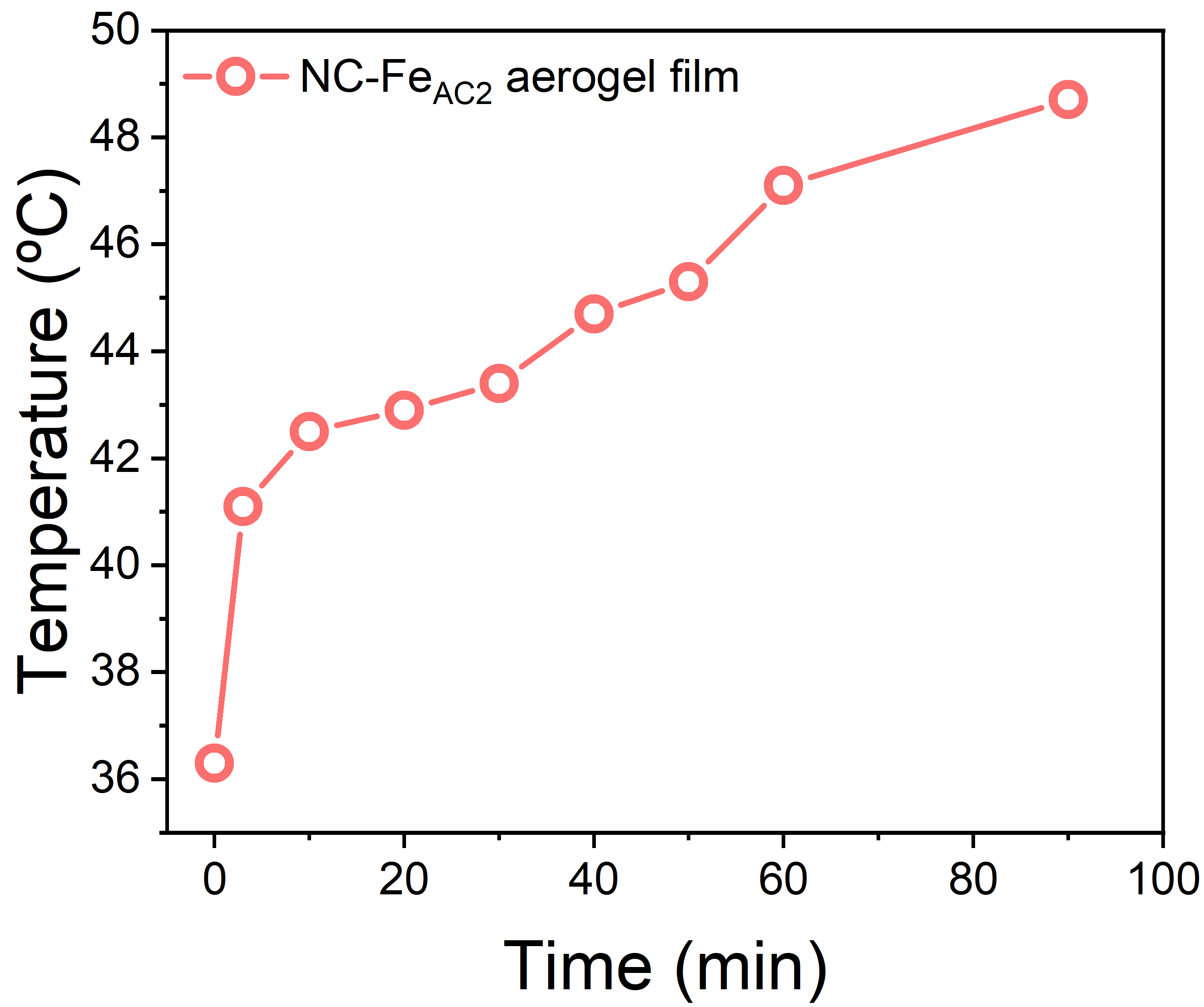


**Fig. S39** Surface temperature variation of the samples with the increasing heating time


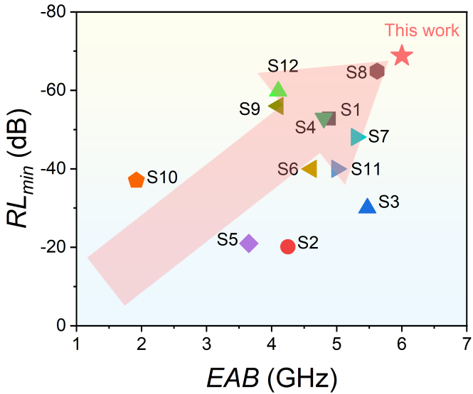


**Fig. S40** Comparison of EMW absorption performance under marine environments [S1-S12]

**Supplementary References**

1. Q. Ban, Y. Song, L. Li, H. Zhang, X. Wu et al., Confined diffusion engineering of FeCoNi-embedded hollow carbon microcage toward controllable electromagnetic wave absorption and anticorrosive polyvinylidene fluoride composite in marine environment. Small **21**(41), e08008 (2025). <https://doi.org/10.1002/smll.202508008>
2. B. Li, L. Ma, S. Li, J. Cui, X. Liang et al., Electromagnetic wave absorption and corrosion resistance performance of carbon nanoclusters/Ce–Mn codoped barium ferrite composite materials. Int. J. Miner. Metall. Mater. **32**(3), 699–709 (2025). <https://doi.org/10.1007/s12613-024-2997-2>
3. J. Yao, J. Zhou, L. Lu, F. Yang, Z. Yao et al., Rare earth lanthanum pinning effect for corrosion resistance ultraefficient microwave absorption FeCo@rGO composites. J. Mater. Sci. Technol. **177**, 181–190 (2024). <https://doi.org/10.1016/j.jmst.2023.07.075>
4. M. Zhao, S. Ran, X. Guo, Z. Wang, Q. Hou et al., Magnetic-dielectric synergy in Fe_3_O_4_/MoS_2_@polyaniline composites for high-efficiency electromagnetic wave absorption and corrosion resistance. J. Alloys Compd. **1048**, 185300 (2025). <https://doi.org/10.1016/j.jallcom.2025.185300>
5. W. Wang, F. Qu, Y. Zhang, Z. Liu, H. Chang et al., Enhanced corrosion resistance of flaky carbonyl iron through dual silane surface modification for the application of electromagnetic wave absorption coatings. J. Mater. Sci. **59**(4), 1721–1735 (2024). <https://doi.org/10.1007/s10853-023-09301-w>
6. X. Zhao, H. Li, W. Chen, W. Li, Z. Han et al., Carbon-based composites from Ti-Ni MOFs with enhanced electromagnetic wave absorption and corrosion resistance. Colloids Surf. A Physicochem. Eng. Aspects **727**, 138369 (2025). <https://doi.org/10.1016/j.colsurfa.2025.138369>
7. H. Wang, H. Ren, C. Jing, J. Li, Q. Zhou et al., Two birds with one stone: Graphene oxide@sulfonated polyaniline nanocomposites towards high-performance electromagnetic wave absorption and corrosion protection. Compos. Sci. Technol. **204**, 108630 (2021). <https://doi.org/10.1016/j.compscitech.2020.108630>
8. L. Duan, J. Zhou, J. Tao, Y. Liu, Y. Yan et al., Genome engineering of materials based on Ce doping, high-performance electromagnetic wave absorber for marine environment. Compos. Part B Eng. **287**, 111882 (2024). <https://doi.org/10.1016/j.compositesb.2024.111882>
9. J. Ge, Y. Cui, L. Liu, F. Meng, F. Wang, The fabrication of FeMnO/RGO as anticorrosive microwave absorbent toward marine environment. Synth. Met. **282**, 116933 (2021). <https://doi.org/10.1016/j.synthmet.2021.116933>
10. H. Liu, W. Xu, H. Ren, D. Li, J. He et al., Integrated comprehensive protection coating achieved by ligand engineering modulated MXene@LDH heterojunction with anti-corrosion, electromagnetic wave absorption and fire safety. Chem. Eng. J. **486**, 150444 (2024). <https://doi.org/10.1016/j.cej.2024.150444>
11. P. Liu, Z. He, X. Li, L. Ding, S. Liu et al., Multifunctional hollow carbon microspheres enable superior electromagnetic wave response and corrosion barrier. Adv. Mater. **37**(35), 2500646 (2025). <https://doi.org/10.1002/adma.202500646>
12. Y. Wang, S. Zhu, H. Guan, Q. Man, Silica coated MOFs-derived composites with excellent electromagnetic wave absorption and corrosion resistance. Colloids Surf. A Physicochem. Eng. Aspects **694**, 134043 (2024). <https://doi.org/10.1016/j.colsurfa.2024.134043>
